# A pan-disease and population-level single-cell TCRαβ repertoire reference

**DOI:** 10.1038/s41421-025-00836-7

**Published:** 2025-10-14

**Authors:** Ziwei Xue, Lize Wu, Bing Gao, Ruonan Tian, Yiru Chen, Yicheng Qi, Tianze Dong, Yadan Bai, Yu Zhao, Bing He, Lie Wang, Zuozhu Liu, Jianhua Yao, Linrong Lu, Wanlu Liu

**Affiliations:** 1 Department of Rheumatology and Immunology of the Second Affiliated Hospital, and Centre of Biomedical Systems and Informatics of Zhejiang University-University of Edinburgh Institute, Zhejiang University School of Medicine, Hangzhou, Zhejiang China; 2 Biomedical Sciences, College of Medicine and Veterinary Medicine, University of Edinburgh, Edinburgh, UK; 3 Institute of Immunology and Department of Rheumatology at Sir Run Run Shaw Hospital, Zhejiang University School of Medicine, Hangzhou, Zhejiang China; 4 Shanghai Immune Therapy Institute, Renji Hospital, Shanghai Jiao Tong University School of Medicine, Shanghai, China; 5 AI for Life Sciences Lab, Tencent, Shenzhen, Guangdong Province China; 6 Bone Marrow Transplantation Center and Institute of Immunology, the First Affiliated Hospital, Zhejiang University School of Medicine, Hangzhou, Zhejiang China; 7 Zhejiang University-Angelalign Inc. R&D Center for Intelligent Healthcare, Zhejiang University-University of Illinois Urbana-Champaign Institute (ZJU-UIUC Institute), International Campus, Zhejiang University, Haining, Zhejiang China; 8 Zhejiang Key Laboratory of Medical Imaging Artificial Intelligence, Haining, Zhejiang China

**Keywords:** Bioinformatics, Immunology

## Abstract

Recent advances in single-cell technology enable the simultaneous capture of T cell receptor (TCR) sequences and gene expression (GEX), providing an integrated view of T cell function. However, linking TCRαβ information and T cell phenotypes at the population level to elucidate their disease association remains an unaddressed gap. Here, by constructing a large-scale reference of paired single-cell RNA/TCR sequencing (scRNA/TCR-seq) comprising more than 2 million T cells from 70 studies, 1017 biological samples, 583 individuals, and 46 disease conditions, along with their single-cell transcriptome, full-length paired TCR, and human leukocyte antigen (HLA) genotypes, we revealed the intrinsic features of germline-encoded TCR-major histocompatibility complex (MHC) restriction in CD4^+^/CD8^+^ lineages. We also observed widely existing public TCRαβs across the population, associated with higher clonal expansion levels and shared HLA alleles. The most publicly shared TCRs are likely to target epitopes from common viruses, such as Epstein-Barr virus (EBV), cytomegalovirus (CMV), and influenza A virus (IAV). Furthermore, we introduced TCR-DeepInsight, a computational framework to identify HLA-shared and disease-associated TCRαβ clusters that exhibit similar TCR sequence and GEX profiles, extensible for researchers to incorporate their data with our reference and characterize potentially functional TCRs. In summary, our work presents a panoramic scTCRαβ reference and computational methods for TCR study.

## Introduction

T cells play a crucial role in the adaptive immune response by recognizing and eliminating pathogen-infected cells and providing cancer immunosurveillance in an antigen-specific manner through the recognition of pMHC (peptide-MHC) via the TCR on the cell surface. TCR consists of α and β chains with highly variable regions on both chains, contributing to the binding specificity between TCRs and antigen peptides. During their development in the thymus, T cells generate an enormous diversity of TCRs through V(D)J recombination and random nucleotide insertions and deletions in both α and β chains, resulting in a theoretically estimated total diversity of TCRs ranging from 10^15^ to 10^61^ before thymic selection^1–3^ and 10^6^ to 10^8^ in young adults after selection^4–6^, enabling the recognition of an almost infinite number of pathogen-derived or self-antigens.

TCRs with identical variable and junction (V/J) genes and Complementarity Determining Regions 3 (CDR3) amino acid sequences for both α and β chains in different individuals are theoretically rare due to the total combinatorial diversity, while recent studies have continuously emphasized the overlap of TCR repertoire across individuals^7–11^. These TCRs, known as public TCRs, are thought to arise from convergent recombination, recombinatorial biases, thymic selection, and peripheral selection, enabling them to recognize the same antigen epitope presented by shared MHC molecules in different individuals^7,8,10^. In response to infections, cancer progression, or autoimmune diseases, T cells undergo clonal expansion, resulting in populations of T cells with identical TCRs and shared antigen specificity. These clonally expanded T cells can exhibit substantial heterogeneity in gene expression and transcriptional programs, reflecting diverse activation states and effector functions. Among these TCRs, public TCRs often represent a special and infrequent subset recognizing conserved antigens associated with specific pathological conditions. Such TCRs may provide insights into the identification of disease-associated TCRs and contribute to the development of future TCR-T immunotherapies. Given the vast diversity of TCRs and T cell phenotypes, there is a pressing need to identify additional public or disease-associated TCRs at the population level.

Single-cell immune profiling technologies enable the simultaneous measurement of both paired TCRαβ repertoires and GEX profiles at single-cell resolution, allowing linking individual T cell clonotypes to their transcriptomic phenotypes during immune responses^12–14^. Computational methods, including GLIPH/GLIPH2^15,16^, TCRdist/TCRdist3^17,18^, GIANA^19^, iSMART^20^, and ClusTCR^21^, are designed to cluster large-scale TCR repertoire datasets based on TCR sequence similarity. In addition to TCR information, recent approaches, including CoNGA^22^, Tessa^23^, scNAT^24^, mvTCR^25^, and MIST^26^ incorporate transcriptomic information from single-cell RNA sequencing (scRNA-seq) to jointly represent TCR and GEX. While these methods have primarily focused on analyses involving limited datasets, individuals, or disease conditions, the incorporation of comprehensive and population-level disease and HLA information with TCR and GEX is critical for identifying functional TCRs. Current approaches, however, often fail to provide a panoramic view of the scTCRαβ repertoire landscape.

To tackle the outlined challenges, we curated population-level single-cell immune profiling datasets of CD8^+^ and CD4^+^ T cells from various disease conditions with full-length TCRαβ chains, single-cell transcriptome, and Human Leukocyte Antigen (HLA) genotype. We developed a computational framework, TCR-DeepInsight, to jointly represent GEX profiles and TCR sequences, with an embedded HLA and disease association scoring function to aid the characterization of functional TCRs. We demonstrated that our population-level single-cell TCRαβ (scTCRαβ) reference, along with TCR-DeepInsight, identifies the immense existence of TCRαβ clonotypes with convergent TCR amino acid sequence and similar GEX profiles among populations, with shared HLA genotypes and association with particular disease conditions. Moreover, our pre-trained model enables the transfer of expanding T cell immune-profiling datasets to our reference, facilitating cross-population and pan-disease comparison, and the identification of disease-associated TCRαβ clusters.

## Results

### Human T cell paired TCRαβ repertoire data collection and integration

**Fig. 1:**
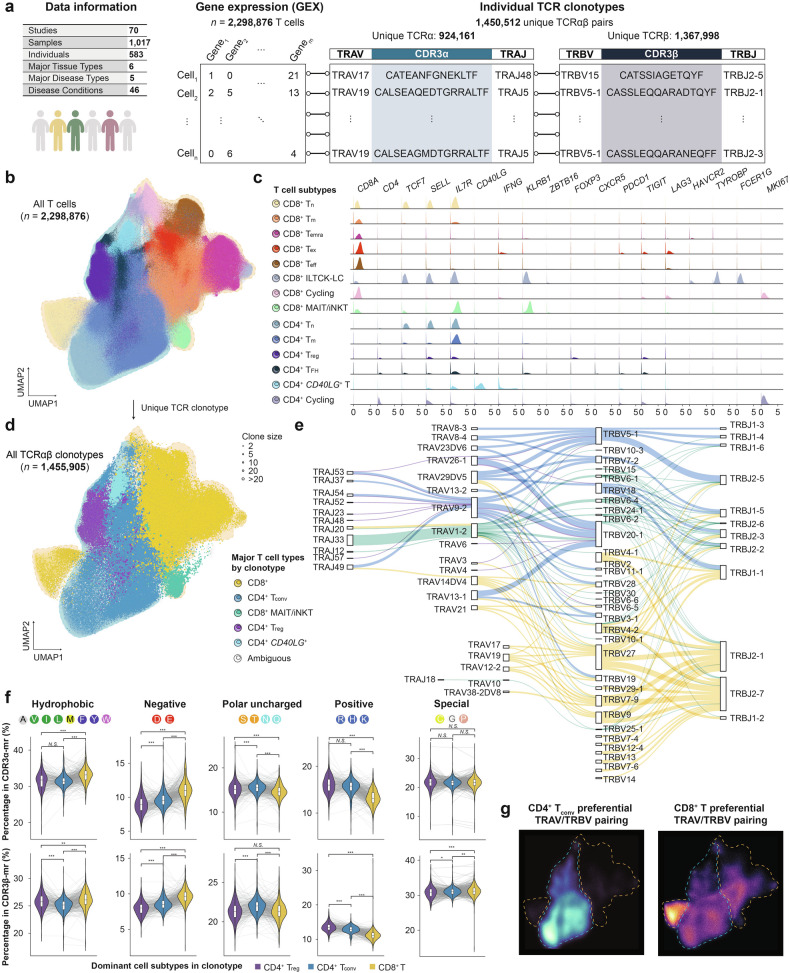
Overview of the pan-disease single-cell TCRαβ repertoire reference atlas. **a** Pan-disease and population-level data collection of scRNA/TCR-seq data. **b** UMAP of the integrated datasets colored by T cell subtypes. **c** Kernel density estimation plot of expression of key marker genes for T cell subtypes. **d** UMAP of the unique clonotypes displaying corresponding single-cell transcriptome information colored by the T cell types. **e** Sankey plot illustrating the enriched TRAV/TRAJ, TRAV/TRBV, and TRBV/TRBJ pairing and joining preferences in major T cell types. **f** Violin plot showing the preference for amino acids with different physicochemical properties in the CDR3 middle region (mr) of CD4^+^ T_reg_, CD4^+^ T_conv_, and CD8^+^ T cells. Each gray dot in the plot represents data from one individual, while lines connecting gray dots indicate amino acid usage differences in different cell types within each individual, considering support from at least 100 cells. White dots within the violin plot represent the average percentage of amino acid usage. Letters within colored dots represent the various physicochemical properties of amino acids. **g** UMAP view of relative cell density plot of clonotypes either using CD4^+^ T_conv_ (left) or CD8^+^ T (right) preferential TRAV/TRBV. The blue and orange dashed lines outline the CD4^+^ T_conv_ and CD8^+^ T cells defined by gene expression. ***P < 0.001, **P < 0.01, *P < 0.05, paired *t*-test; *N.S*., not significant.

Our continuous efforts in the collection of T cell immune profiling datasets expanded the human Antigen Receptor database^27^ (huARdb, https://huarc.net/v2/database/) to include 70 studies and 1017 biological samples from 583 individuals and 46 disease conditions, including solid tumor, leukemia, inflammation/autoimmune, infections, and healthy (Fig. 1a; Supplementary Fig. S1a and Table S1). With datasets collected in huARdb, we have previously developed an atlas-level integration tool scAtlasVAE and established a human CD8^+^ T cell reference atlas, and analyzed the phenotypic transition among different CD8^+^ T cell subtypes^28^. In this study, we further expanded our datasets to include CD4^+^ T cells, encompassing a total of 2,298,876 high-confidence T cells with paired transcriptome and full-length α/β TCR information. This enabled us to perform a large and unbiased analysis of the scTCRαβ repertoire across diseases and individuals. These datasets contain 1,450,512 unique TCRαβ clonotypes, with 924,161 unique TCRα chains defined by the same T cell receptor alpha variable gene (TRAV)-CDR3α-T cell receptor alpha joining gene (TRAJ), and 1,367,998 unique TCRβ chains defined by the same T cell receptor beta variable gene (TRBV)-CDR3β-T cell receptor beta joining gene (TRBJ) (Fig. 1a). HLA genotypes for each individual were determined from the single-cell transcriptome dataset using arcasHLA, a state-of-the-art method for HLA genotyping in scRNA-seq^29,30^ (Supplementary Fig. S1a–c). In addition, we established a comprehensive bulk reference dataset comprising over 60 million unique TCRβ sequences from TCRdb^31^ and the immuneACCESS database (Supplementary Fig. S1a and Table S2). To enable rapid query of TCRα, β, or TCRαβ pairs, we developed a web application for searching TCRs with similar sequences across single-cell and bulk TCR datasets, with curated disease information (https://huarc.net/v2/search/).

To gain an accurate cellular phenotype in the single-cell data, we integrated the gene expression (GEX) modality of the datasets using scAtlasVAE^28^ and obtained a latent embedding of transcriptome features representing T cell subtype, with a harmonized distribution of studies compared to embeddings from principal component analysis (PCA) (Fig. 1b; Supplementary Fig. S2a). We categorized T cells into CD8^+^ and CD4^+^ cell types, aligned with the expression pattern of key marker genes, and showed varying composition in different tissue types and disease types (Fig. 1c; Supplementary Fig. S2b–d). The CD8^+^ cell type was further categorized into naïve (CD8^+^ T_n_), memory (CD8^+^ T_m_), recently activated effector memory (CD8^+^ T_emra_), exhausted (CD8^+^ T_ex_), effector (CD8^+^ T_eff_), cycling, innate-like T cell with high cytotoxic potential-like cell (CD8^+^ ILTCK-LC), and innate-like T cells that recognize non-peptide antigens, including mucosal invariant T cells and invariant natural killer T cells (CD8^+^ MAIT/iNKT). The CD4^+^ cell includes naïve (CD4^+^ T_n_), memory (CD4^+^ T_m_), regulatory (CD4^+^ T_reg_), follicular helper (CD4^+^ TFH), CD4^+^*CD40LG*^+^ T, and cycling T cells. Using GEX-based subtype annotations, we assigned each clonotype to a major T cell type, including conventional CD4^+^ T cells (CD4^+^ T_conv_), CD4^+^ T_reg_, CD8^+^ T, CD8^+^ MAIT/iNKT, and CD4^+^*CD40LG*^+^ T cells (Fig. 1d). To our knowledge, this is so far the most comprehensive pan-disease scTCR repertoire reference with paired TCRαβ sequences and transcriptome profile, along with HLA genotype and disease information for each individual.

### Analysis of TCR intrinsic features in CD4^+^ and CD8^+^ T cells

During thymic positive selection, TCRαβ recognizes MHC class I or class II, driving the T cell to differentiate into CD4^+^ or CD8^+^ T cells. Despite the existence of cross-reactive TCR to MHC class I and class II and overlap of TCRαβ repertoire between CD4^+^ and CD8^+^ populations, we found limited overlap between CD4^+^ and CD8^+^ repertoire in our population-level TCRαβ repertoire^32^ (Supplementary Fig. S3a). These findings highlight the association between the intrinsic TCRαβ, MHC class I or class II restriction, and T cell lineage commitment^33,34^.

The transcriptomic information of our population-level TCRαβ repertoire allows us to analyze the association of V/J gene usage and CDR amino acid preference among different T cell types. As expected, MAIT cells show the most biased joining between TRAV and TRAJ genes, consistent with the well-established observation that they preferentially use TRAV1-2 paired with TRAJ33, TRAJ12, and TRAJ20^35^, and iNKT cells use TRAV10 and TRAJ18 to form their invariant α chains^36,37^ (Fig. 1e). For β chain, MAIT cells preferentially select TRBV6-1, TRBV6-2, TRBV6-4, and TRBV20-1, and iNKT cells select TRBV25-1 as reported before^38,39^ (Fig. 1e). The restricted usage of V/J genes observed in both the TCR α and β chains of MAIT cells is attributed to their recognition of the MHC-like molecule MR1^40^. Preferential TRAV/TRBV pairing was observed for CD8^+^ T and CD4^+^ T_conv_ clonotypes, where TRAV14DV4/TRBV27 and TRAV23DV6/TRBV5-1 are prominently biased TRAV/TRBV pairings for CD8^+^ T and CD4^+^ T_conv_ clonotypes, respectively (Fig. 1e, g), providing additional evidence to previous single-chain analysis^32,41,42^.

As the CDR1 and CDR2 regions often contact the conserved α-helices of MHCs^43,44^ and the CDR3 region frequently recognizes MHC class I or class II displayed peptide, we asked whether the CDR regions may display different biochemical properties in T cell types, including CD4^+^ T_reg_, CD4^+^ T_conv_, and CD8^+^ T cells. We find limited amino acid length difference for either CDR3α/β or the somatically generated CDR3α/β middle region (CDR3-mr) in these T cell types, suggesting that the length of the CDR3-mr may not contribute to T cell lineage specification (Supplementary Fig. S3b). MAIT cells used shorter CDR3α or CDR3α-mr due to their relatively invariant α chain usage length with 12 amino acids (Supplementary Fig. S3c,d).

Previous reports showed the preference of hydrophobic amino acid usage in the CDR3β-mr in CD4^+^ T_reg_ compared to CD4^+^ T_conv_ in both humans and mice^45,46^, and discriminated usage of positively charged amino acid in CD8^+^ T cells compared to CD4^+^ T_conv_ in mouse models^47^, which is also observed in our population-level data, independently of HLA genotype (Fig. 1f). In our analysis, we further showed a higher proportion of hydrophobic and negatively charged and lower proportion of positively charged amino acids in CD8^+^ T cells compared to CD4^+^ T cells, and a similar pattern was observed in CDR3α-mr (Fig. 1f). We also found preferential usage of amino acids with certain biochemical properties in CDR1 and CDR2 region for both α and β chains among CD8^+^ T, CD4^+^ T_reg,_ and CD4^+^ T_conv_ cells, possibly reflecting that the V genes jointly contribute to germline-encoded recognition of class I and class II MHC^48–50^ (Supplementary Fig. S4a). The amino acid usage preference observed in scTCRβ repertoire can largely be resembled by bulk datasets, indicating that these phenomena are likely to be a general feature (Supplementary Fig. S4b).

### HLA-sharing and clonal expansion serve as the major determinants for public TCRαβs

**Fig. 2:**
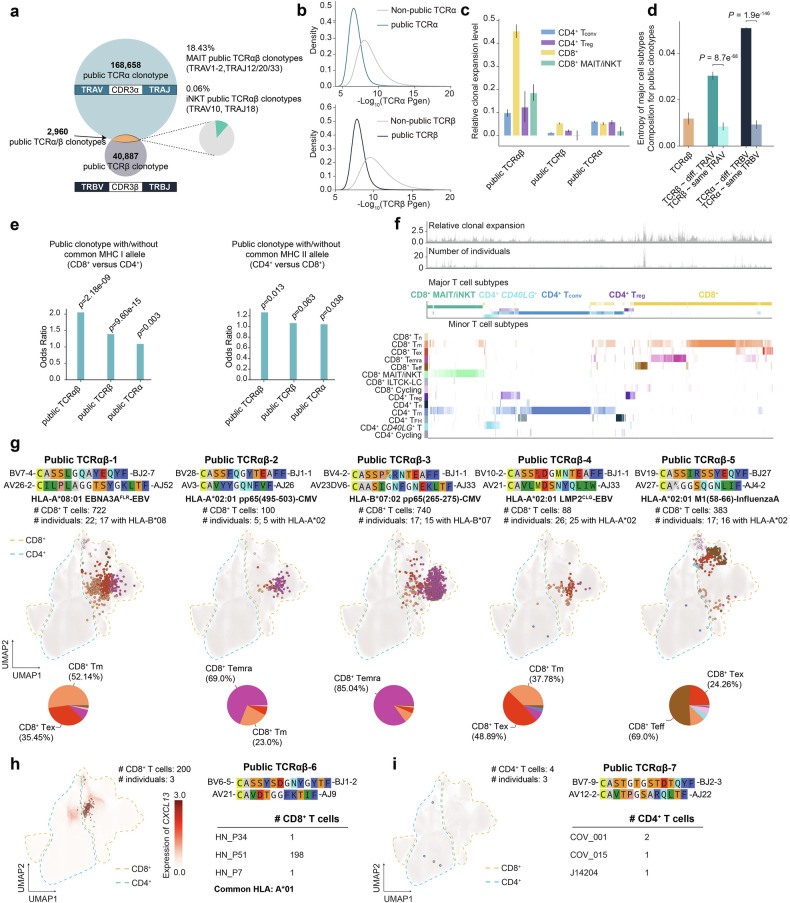
Analysis of the public TCRs from the large-scale scTCR-seq reference atlas. **a** Overview of public TCRs defined by the same alpha chain (TRAV, CDR3α, TRAJ), beta chain (TRBV, CDR3β, TRBJ), or both. **b** Distribution of TCR generation probabilities for public and non-public TCRα and TCRβ sequences. **c**, **d** Bar plots showing relative clonal expansion levels defined by the log10-transformed ratio of number of cells to the number of public clonotypes (**c**), and Shannon entropy of major cell subtype distributions (**d**) for public TCRαβ, TCRα, and TCRβ. Error bars show the 95% confidence intervals. Statistical comparisons of Shannon entropy between public TCRα and TCRβ with identical or distinct V gene pairings were performed using a *t*-test. **e** Number of public clonotypes predominantly composed of CD8^+^ or CD4^+^ T cells shared with the same class I or class II HLA genotypes, represented by odds ratios (OR) and assessed via Fisher’s exact test. **f** Overview of clonal expansion (top), number of individuals contributing (middle), and major/minor cell subtype composition (bottom) for public TCRαβ clonotypes. **g** UMAP visualization of representative public TCRαβ clonotypes with known antigen specificity and convergent gene expression, with accompanying cell subtype composition in pie charts. **h** UMAP of *CXCL13* expression and analysis of shared beta chain sequences in a bulk dataset for a public TCRαβ clonotype of unknown antigen specificity in three HNSCC patients. **i** UMAP depicting a public TCRαβ clonotype exclusive to COVID-19 patients in both scTCR-seq and bulk TCR-seq data.

In our massive paired and full-length TCRαβ collection, we identified 2960 public TCRαβ clonotypes (with identical TRAV-CDR3α-TRAJ, and TRBV-CDR3β-TRBJ), 168,658 public TCRα clonotypes (identical TRAV-CDR3α-TRAJ), and 40,887 public TCRβ clonotypes (identical TRBV-CDR3β-TRBJ) (Fig. 2a; Supplementary Tables S3–S5). The overlap is more prevalent in the non-naïve repertoire than the naïve repertoire, suggesting the effect of peripheral selection in TCR publicness (Supplementary Fig. S5a). Given the comparable number of total unique TCRα and TCRβ in the dataset, the significantly higher number of public TCRα indicates a generally more conserved α chain repertoire across populations (Fig. 2a). Furthermore, a substantial fraction of TCRβ chains identified in all TCRαβ clonotypes (65.75%), public TCRαβ clonotypes (81.96%), and public TCRβ clonotypes (92.88%) were also detected in bulk sequencing data, indicating that TCR publicness may be more widespread than anticipated (Supplementary Fig. S5b).

To understand potential characteristics contributing to TCRαβ publicness, we annotated each TCRα or TCRβ sequence with the generation probability calculated by OLGA^51^ using the CDR3 amino acid sequence and V/J genes. We found that public TCRα and TCRβ clonotypes were all associated with significantly higher generation probability^52,53^ (Fig. 2b). Public TCRαβs are more clonally expanded compared to public TCRα or TCRβ clonotypes, especially in CD8^+^ T cells, indicating their conserved functional roles across populations^52^ (Fig. 2c). Interestingly, cell subtype composition of public TCRαβ clonotypes in different individuals is more consistent compared to public TCRα or TCRβ clonotypes, suggesting that the T cell phenotype is likely to be determined by both α and β chains rather than by either chain alone (Fig. 2d). In addition, when public TCRα is paired with the same TRBV, the resulting clonotypes exhibit more homogeneous cell type composition compared to those paired with different TRBVs. Similarly, this pattern is observed for public TCRβ, suggesting that T cell lineage commitment may depend on the usage of V genes from both α and β chains (Fig. 2d). We further demonstrated that the cell type composition is more heterogeneous when a given TCRα and TCRβ pair with diverse V genes (Supplementary Fig. S5c, f). Specifically, this phenomenon could be exemplified by several public TCRαs and TCRβs, which could pair with the same or different V genes and displayed convergent or distinct CD4^+^ and CD8^+^ phenotypes, respectively (Supplementary Fig. S5d, e, g, h).

Moreover, compared to TCRα or TCRβ clonotypes, public TCRαβ clonotypes are significantly associated with shared class I or class II HLA alleles across individuals, contingent on whether the predominant cell subtype within the clonotype is CD4^+^ or CD8^+^ (Fig. 2e), suggesting that public TCRs are likely shaped through an MHC-dependent manner^54^. These findings collectively indicate that the TCR α and β chains work collaboratively to facilitate MHC recognition and T cell lineage commitment at a population level.

### Public TCRαβ repertoire and T cell states are shaped by common viral infection

We hypothesized that public TCRs in different individuals may bind to shared epitopes, and T cells harboring these T cell receptors exert similar cytotoxic or memory functions across populations. We first asked whether public TCRαβ clonotypes would bind epitopes derived from common viruses such as Epstein-Barr virus (EBV), Cytomegalovirus (CMV), and Influenza A virus (IAV), as they are previously reported to trigger public T cell response^10,11^. We identified a public and clonally expanded public clonotype TCRαβ-1 from 22 individuals, whose CDR3β has previously been reported to recognize HLA-A*08:01-restricted EBV-derived EBNA3A^FLR^ epitope^55^ (Fig. 2g). A majority of T cells from this public clonotype are CD8^+^ T_m_, indicating a robust and long-lasting immune response against this epitope (Fig. 2g). Another clonally expanded public TCRαβ-2 clonotype recognizing HLA-A*02:01-restricted CMV-derived pp65(495**–**503) epitope^15,56,57^ was found in 5 individuals, and most T cells in the clonotype are annotated as CD8^+^ T_emra_, suggesting that this clonotype may be associated with a potent and immediate effector response upon antigen exposure in various individuals (Fig. 2g).

Interestingly, several public TCRαβ clonotypes only differ by a single amino acid in CDR3β and share similar transcriptomic profiles across populations. For example, in the public clonotype TCRαβ-3, both public clonotypes P(S/Q)R CDR3β motif have been reported to recognize HLA-B*07:02-restricted CMV-derived pp65(265**–**275) epitope^11,15,58^, and their corresponding T cells are majorly CD8^+^ T_emra_ (Fig. 2g). In other examples, we identified public TCRαβ clonotypes that differ by only a single amino acid in either the CDR3β (public TCRαβ-4) or CDR3α (public TCRαβ-5) regions (Fig. 2g). Specifically, within the public clonotype TCRαβ-4, one clonotype containing the EDG CDR3β motif shares the TCRβ with a TCR that recognizes the HLA-A*02:01*-*restricted EBV-derived LMP2^CLG^ epitope^59^. In the public clonotype TCRαβ-5, both clonotypes share the TCR β chain with a TCR that targets the HLA-A*02:01-restricted M1(58**–**66) epitope from the IAV^60^. Given the conserved CDR3 motif, convergent T cell phenotype, and shared HLA alleles among individuals, we speculate that these TCRαβ may recognize the same epitope.

Nevertheless, only 33.24% of the public TCRαβ clonotypes are associated with a single disease type, with the majority being associated with COVID-19 and solid tumors (Supplementary Fig. S5i, j). The public TCRαβ-6 clonotype was discovered in three head and neck squamous carcinoma (HNSCC) patients (Fig. 2h). This clonotype is shared among T cells with highly similar transcriptome profiles in CD8^+^ T_ex_ cells, which highly express *CXCL13*, suggesting their potential tumor-reactive function^61^, while this TCRβ chain was also found in more than ten individuals in the bulk dataset with no incidence of solid tumors (Supplementary Table S3). Another representative example is the public TCRαβ-7 clonotype found in three individuals with COVID-19 infection, with the β chain also identified in two individuals with COVID-19 in the bulk dataset (Fig. 2i). Although there are no previous reports on this specific clonotype, it may recognize a class II HLA-restricted epitope derived from SARS-CoV-2 due to their CD4^+^ phenotype.

### Development of TCR-DeepInsight to jointly represent TCR and GEX

One of the main focuses in TCR repertoire analysis is to identify TCR clusters with a higher probability of recognizing the same antigen peptide. Previous studies and our analysis suggest that V genes and CDR3 amino acid sequence from both TCR α and β chains jointly contributed to the T cell phenotype and epitope specificity^62^. Moreover, it has been recently emphasized that incorporating TCR sequence and transcriptome information from single-cell data can enhance the clustering of TCRs with the same antigen specificity^22,23,25,63^.

**Fig. 3:**
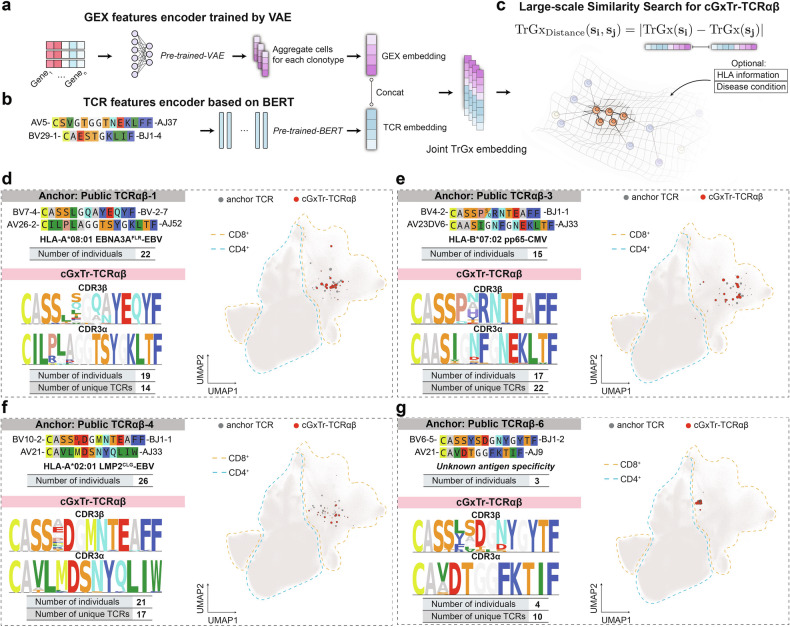
Searching for TCRαβ clonotypes with convergent TCR and GEX by representation learning. **a**–**c** Schematic of the joint GEX/TCR representation learning approach: VAE for GEX (**a**) and BERT for TCR sequence features (**b**), followed by concatenation of embeddings from both GEX and TCR data for large-scale similarity searches in the joint representation space (**c**). **d**–**g** Examples of cGxTr-TCRαβ clonotypes using similarity searches anchored by two public TCRαβ clonotypes with known antigen specificity (**d**–**f**) and one clonotype with unknown specificity (**g**). Motif plots of CDR3α and CDR3β from the cGxTr-TCRαβ clusters and the UMAP positions of their corresponding cells are displayed.

To robustly cluster TCRαβ considering both TCR sequence similarity and transcriptome features from a million-level paired TCRαβ repertoire, we developed a deep-learning-based framework named TCR-DeepInsight. We first extracted the latent embedding learned by scAtlasVAE, a variational autoencoder model, to represent the GEX features with the removal of batch effects from population-level scRNA-seq data (Fig. 3a).

In parallel, to learn the underlying relationship for amino acids within the CDR1/2/3 region from both TCR α and β chains, we adopted the Bidirectional Encoder Representations from Transformers (BERT) (Fig. 3b), which has been shown to be effective with biological sequence data, including TCR amino acid sequences^64–67^. We used 1,455,905 unique TCRαβ sequences to pretrain an unsupervised BERT model by randomly masking amino acids within the CDR1/2/3 region of either α and β chains and obtained the TCR embeddings.

To match the TCR embedding, we averaged GEX embeddings for each TCRαβ clonotype based on its originating cells, resulting in an aggregated GEX embedding (Fig. 3a). We subsequently combined the TCR embedding with the aggregated GEX embedding to construct a TCR-GEX (TrGx) joint embedding of TCRαβ at the population level. The Euclidean distance within this joint embedding (referred to as TrGx distance) was used as a metric to assess the similarity for both TCR sequences and transcriptomic features (Fig. 3c).

### TrGx joint embedding facilitates TCRαβ clustering with convergent TCR and GEX

A positive correlation was observed between the Levenshtein distance of CDR3α and CDR3β amino acid sequences and the TrGx distance (Supplementary Fig. S6a,b). Additionally, randomly selected TCRαβ clonotypes sharing the same V gene or originating from the same cell type exhibited lower TrGx distances (Supplementary Fig. S6c–e). These findings indicate that the TrGx embeddings effectively capture the similarity of TCR amino acid sequences, V gene usage, and T cell phenotypes within the latent space. Therefore, clustering TCR clonotypes based on the TrGx embeddings integrates both TCR sequence and transcriptomic profiles, enabling dual-modality analysis.

To identify convergent TrGx (cTrGx)-TCRαβ clusters, the TCR-DeepInsight model takes a TCRαβ sequence as an input anchor and groups it with TCRαβ neighbors with the most similar k value (where k is a predefined number) based on the TrGx distance. As a demonstration, we employed the previously identified public TCRαβ-1, TCRαβ-3, and TCRαβ-4 with known antigen specificity for EBV and CMV, as clustering anchors.

These clusters were identified in a larger number of individuals and exhibited shared V gene usage, consistent gene expression patterns, and highly similar TCR sequence motifs that differed only by specific amino acid substitutions at certain positions in the CDR3 regions (Fig. 3d–f). Using public TCRαβ-6 as the anchor, we identified a cTrGx-TCRαβ cluster encompassing another HNSCC patient, and most of these T cells correspond to CD8^+^ T_ex_ (Fig. 3g). Interestingly, we observed a generally less conserved amino acid usage at positions 5 and 6 of CDR3β in cTrGx-TCRαβ (Fig. 3d–f). By using all public TCRαβ as anchors for searching cTrGx-TCRαβ, we further demonstrated that the amino acid at position 5 in both CDR3α and CDR3β may be the most interchangeable for public TCRs without changing T cell phenotype (Supplementary Fig. S6f,g), consistent with a recent investigation which validated that the antigen specificity of a public TCRαβ recognizing HLA-A*02:01-restricted LMP2^FLY^ epitope is resilient of switching amino acid at position 5 of CDR3β^68^. These findings indicate that TrGx embeddings enable the identification of TCRαβ clusters with potentially similar functions across populations.

### Identification of HLA-shared or disease-associated cTrGx-TCRαβ clusters

**Fig. 4:**
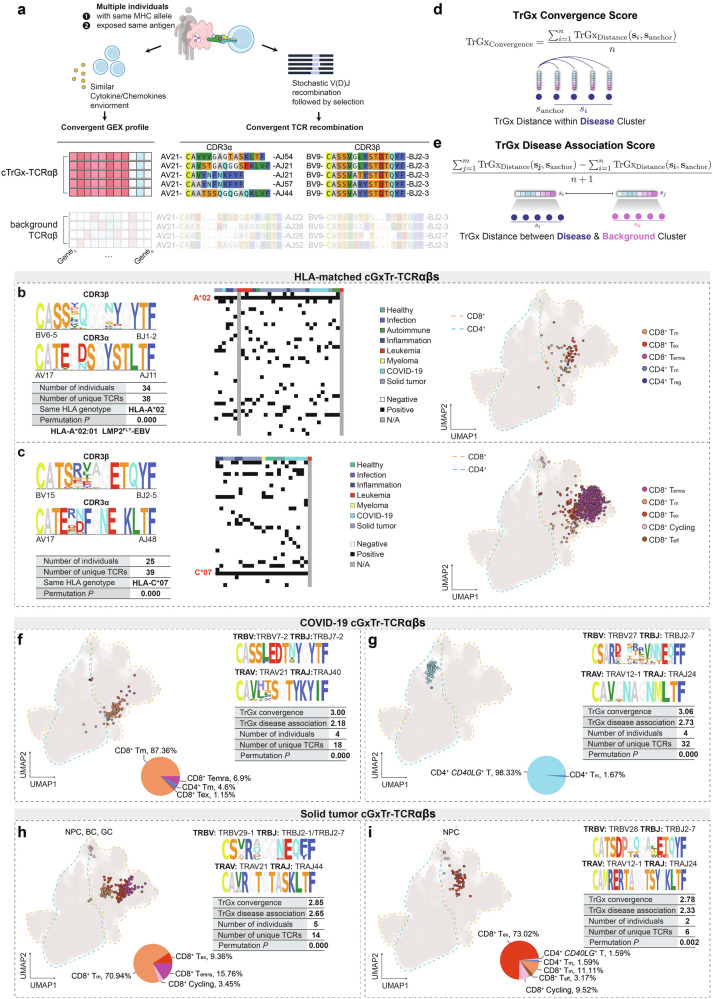
Identification of HLA-shared and disease-associated cTrGx-TCRαβ clonotype clusters. **a** Overview of cGxTr-TCRαβ clonotypes characterized by convergent TCR sequences and gene expression profiles. **b**, **c** Representative cGxTr-TCRαβ clonotypes clustered by matching HLA-A*02 with known antigen specificity (**b**) and HLA-C*07 with unknown antigen specificity (**c**). **d**, **e** Definitions of TrGx convergence score (**d**), and TrGx disease association score (**e**). The binary heatmaps shows the HLA genotypes from all individuals for each cluster. **f**, **g** Examples of COVID-19-associated cGxTr-TCRαβ clonotypes predominantly composed of CD8^+^ Tm cells (**f**), CD4^+^*CD40LG*^+^ T cells (**g**). **h**, **i** Examples of solid tumor-associated cGxTr-TCRαβ clonotypes dominated by CD8^+^ Tm cells (**h**) and CD8^+^ T_ex_ cells (**i**). The number of individuals, number of unique TCRαβs, and *P* values from permutation tests are labeled for each cGxTr-TCRαβ cluster. For **d**–**i**, motif plots of CDR3α and CDR3β from the cGxTr-TCRαβ clusters and the UMAP positions of their corresponding cells are displayed.

The widespread existence of cTrGx-TCRαβ clusters could be attributed to the same thymic selection processes on the TCR repertoire from multiple individuals with shared HLA alleles and epitopes, and activation of T cells with these TCRαβ (Fig. 4a). Despite the degree of public TCRαβ in the naïve repertoire does not have to be dependent on HLA matching as a result of “convergent evolution”^9^, clonally expanded public TCRαβs in memory T cells are likely to recognize the same pMHC from different individuals. We therefore attempted to identify cTrGx-TCRαβ clusters with shared HLA alleles in the population-level dataset using TCR-DeepInsight.

We iteratively selected unique TCRαβ clonotypes as anchors and retained only the nearest TCRαβ clonotype that shared at least one HLA allele, defining it as an HLA-shared cTrGx-TCRαβ cluster. For the most common HLA alleles in the population, such as HLA-A*02, HLA-A*11, HLA-A*24, HLA-B*08, and HLA-C*07, the largest cTrGx-TCRαβ clusters with shared HLA alleles were predominantly comprised of CD8 T_m_ cells (Fig. 4b, c; Supplementary Fig. S7). These cTrGx-TCRαβ clusters either contain TCRαβ clonotypes whose TCRβ chains have known antigen specificity, including the public TCRαβ recognizing HLA-A*02:01-restricted LMP2^FLY^ epitope^68^ (Fig. 4b) and BMLF1280 epitope (Supplementary Fig. S7a), and HLA-A*11:01-restricted EBNA3B epitope (Supplementary Fig. S7b), or their pMHC target specificity remains unknown (Fig. 4c; Supplementary Fig. S7c,d).

However, most of these cTrGx-TCRαβ clusters do not necessarily correlate with specific disease types, as they may recognize epitopes derived from common viruses. Therefore, it is necessary to develop a computational method to identify potentially disease-associated cTrGx-TCRαβ clusters from large-scale and population-level single-cell immune repertoire data.

Building on the iterative neighbor search described above, we employed an alternative strategy. Specifically, when an anchor TCRαβ and its closest neighbors originated from the same disease, we defined them as a disease-associated cTrGx-TCRαβ cluster. The same number of next-ranked TCRαβs were designated as background TCRαβ clusters (Fig. 4a). To assess the within-cluster similarity of TCR and GEX, the TrGx distance within disease clusters was calculated and referred to as the TrGx convergence score (Fig. 4d). The TrGx distance between disease-associated cTrGx-TCRαβ clusters and background TCRαβ clusters was defined as the TrGx disease-association score (Fig. 4e). A lower TrGx convergence score indicates higher similarity for TCR and GEX in cTrGx-TCRαβ clusters, and higher TrGx disease-association score reflects a stronger enrichment of a specific disease condition relative to the background. cTrGx-TCRαβ clusters with two unique TCRαβ clonotypes exhibited lower TrGx convergence scores, possibly caused by random events of the same TCRαβ recombination in two individuals (Supplementary Fig. S8a). To access the significance of the disease association, we implemented a statistical assessment using the random permutation test (Materials and Methods). Additionally, disease-associated cTrGx-TCRαβ clusters dominated by MAIT cells generally showed lower TrGx disease-association scores, reflecting their common presence in healthy individuals as a result of their roles in innate-like antimicrobial reactivity, rather than their association with specific disease conditions^69^ (Supplementary Fig. S8b). Therefore, disease-associated cTrGx-TCRαβs were defined as clusters involving at least two individuals, containing a minimum of three unique TCRαβ clonotypes, and composed primarily of non-MAIT cell types, with a defined threshold for the TrGx disease-association score.

These clusters were associated with conditions including COVID-19, solid tumors, inflammation, and autoimmune diseases (Supplementary Table S6 and Fig. S8c–f). Using curated TCRαβ antigen specificity information (Materials and Methods; Supplementary Table S7), we found that 82/767 COVID-associated cTrGx-TCRαβ clusters were matched with epitopes from SARS-CoV-2 (Supplementary Fig. S8g), while disease-associated cTrGx-TCRαβ clusters from other disease conditions, including solid tumors, had limited known antigen specificity (Supplementary Fig. S8h).

In COVID-19 patients, we identified a disease-associated cTrGx-TCRαβ cluster, displaying a convergent pattern similar to that associated with the B15_NQK epitope of SARS-CoV-2^70^. This cluster was found in 4 individuals with a total of 18 unique TCRαβ clonotypes in our reference (Fig. 4f). These T cells were primarily CD8^+^ T_m_ cells, indicating long-term protection after COVID-19 infection. Another cluster converged on the CDR3α sequence CAVGNAGNMLTF, paired with diverse CDR3β sequences, and was predominantly composed of CD4^+^*CD40LG*^+^ T cells, suggesting a potential effector role in COVID-19, although its antigen specificity remains unknown (Fig. 4g). In solid tumors, we also identified disease-associated cTrGx-TCRαβ clusters. For instance, one cTrGx-TCRαβ cluster was shared across five individuals with nasopharyngeal carcinoma (NPC), breast cancer (BC), and gastric cancer (GC), and was composed of CD8^+^ T_m_, CD8^+^ T_emra_, and CD8^+^ T_ex_ cells (Fig. 4h). Another representative cTrGx-TCRαβ cluster, predominantly composed of CD8^+^ T_ex_ cells, was identified from 2 NPC patients (Fig. 4i).

### Benchmark of TCR-DeepInsight with other methods for repertoire analysis

Previous TCR clustering methods, such as GLIPH2^16^, GIANA^19^, iSMART^20^, ClusTCR^21^, TCRdist/TCRdist3^17,18^, CoNGA^22^, Tessa^23^, scNAT^24^, mvTCR^25^, and MIST^26^ utilize various inputs, including CDR3α/β sequences, V/J gene usage, or GEX data (Supplementary Fig. S9a). To ensure fair comparison, we employed our million-scale scTCRαβ reference as a standardized dataset for the TCR clustering task. Given that each method requires a specific input format, we curated the dataset to ensure compatibility with each approach. However, due to the excessive memory demands, TCRdist/TCRdist3^17,18^, CoNGA^22^, and Tessa^23^ were unable to process datasets of this size and were therefore excluded from the benchmark analysis.

Our analysis demonstrates that GLIPH2 produces the highest number of TCR clusters, while TCR-DeepInsight generates a moderate number of clusters (Supplementary Fig. S9b). The TCR clusters from TCR-DeepInsight show the highest consistency of V gene usage from both chains and T cell phenotype (Supplementary Fig. S9c–e). Since methods including GLIPH2 and GIANA do not incorporate transcriptome information as input and give inadequate attention to the α chain, TCRαβ clusters from these methods are often derived from both CD4^+^ and CD8^+^ T cells with diverse TRAV usage and CDR3α sequences (Supplementary Fig. S10a,b). Methods such as scNAT^24^, mvTCR^25^, and MIST^26^, which integrate both TCR and GEX information, often exhibit limitations in large-scale datasets exceeding millions of cells. Specifically, their joint embeddings are frequently affected by batch effects in the GEX modality (Supplementary Fig. S11a) and require further refinement to achieve a harmonized distribution of cell subtypes (Supplementary Fig. S11b).

### TCR-DeepInsight facilitates the discovery of disease-associated TCRαβs in novel datasets

**Fig. 5:**
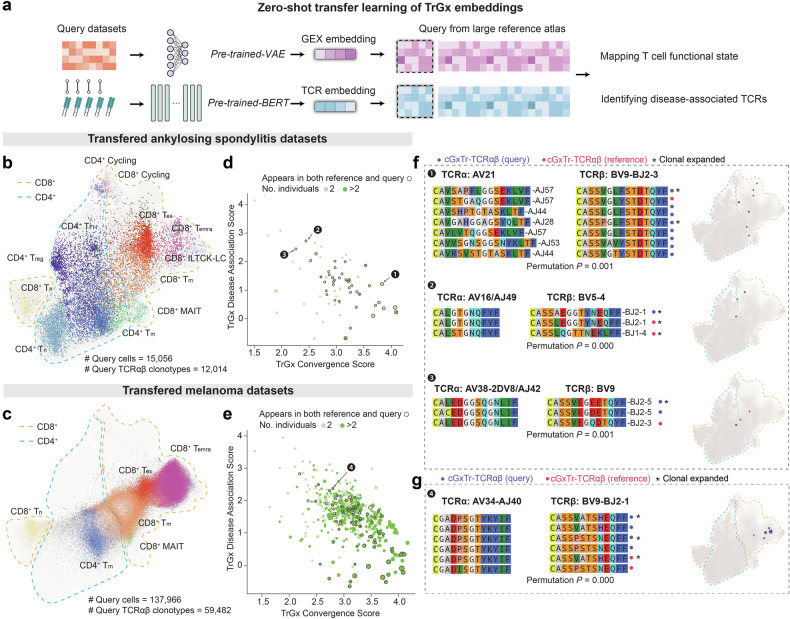
TCR-DeepInsight is extensible for query datasets. **a** Diagram of zero-shot transfer learning for extracting TCR and GEX features. GEX features are obtained using a pretrained VAE model on a reference atlas, while TCR features are derived using a pretrained BERT model on the same reference. **b**, **c** Transferred cell subtype annotations for ankylosing spondylitis (**b**) and melanoma query datasets (**c**). **d**, **e** Scatter plot of TrGx convergence score and TrGx disease association score of clusters of cGxTr-TCRαβ clonotypes associated with ankylosing spondylitis (**d**) and melanoma patients (**e**). **f**, **g** Representative cGxTr-TCRαβ clusters containing clonotypes identified from reference and query datasets of ankylosing spondylitis (**f**) and melanoma patients (**g**). The *P* values from permutation tests are labeled for each cGxTr-TCRαβ cluster.

The pre-trained model from TCR-DeepInsight enables users to rapidly transfer their scTCRαβ datasets to the reference and query for cTrGx-TCRαβ clusters. To demonstrate the transfer performance for TCR-DeepInsight on query datasets, we leveraged additional scRNA-seq and scTCR-seq datasets to identify unique clusters of TCRαβ pairs associated with ankylosing spondylitis (AS)^71^ and melanoma^72^. In parallel with the previously described transfer of pre-trained weights from a larger reference to new query datasets in the scAtlasVAE model^28^, the query TCR embedding can be transferred using the pre-trained BERT model employed for the reference dataset (Materials and Methods, Fig. 5a).

We observed that our query datasets could be projected into a common low-dimensional space with our reference GEX embeddings, with transferred cell subtype annotations (Fig. 5b,c). By applying the aforementioned clustering strategy, we identified cTrGx-TCRαβ clusters specific for AS patients in both reference and query datasets (Fig. 5d). We found that the cTrGx-TCRαβ cluster converges to TCRβ TRBV9/CASSVGLFSTDYQYF/TRBJ2-3, paired with TRAV21 and diverse CDR3α sequence in the CD8^+^ T_m_ and CD8^+^ T_eff_ clusters (Fig. 5f). This CDR3β motif has been previously reported to recognize HLA-B*27-restricted self-antigen or microbial endogenous peptides^73,74^. We also found other cTrGx-TCRαβ clusters associated with AS, yet with unknown antigen specificity, while their disease association with AS required further investigations (Fig. 5f).

For melanoma, we found a cTrGx-TCRαβ cluster observed in 3 melanoma patients involving both query and reference datasets (Fig. 5e, g). It resides in CD8^+^ T_emra_ populations, suggesting its potential involvement in tumor-specific immune responses (Fig. 5g). Interestingly, in the background TCRαβs for this cluster, an expanded TCRαβ clonotype with identical V/J gene usage and CDR3 amino acid sequence for both chains was detected in both the peripheral blood and synovial fluid of a patient who developed inflammatory arthritis following immune-checkpoint inhibitor therapy for melanoma^75^. This observation highlights a shared TCR signature across distinct immune microenvironments, potentially linking anti-tumor immune activity with off-target immune effects.

## Discussion

Advancements in single-cell immune profiling technologies have generated extensive single-cell GEX and TCR datasets, offering tremendous potential to investigate T cell biology and identify functional TCRs. In this study, we present the largest scTCRαβ repertoire reference to date, encompassing over two million CD4^+^ and CD8^+^ T cells derived from 583 individuals spanning 46 disease conditions. This unprecedented scale and diversity enabled us to investigate TCR intrinsic features at the population level and uncover public TCRαβs with shared HLA alleles, convergent gene expression profiles, and antigen specificity information. The broad coverage of different disease conditions and the TCR-DeepInsight method provide a unique resource for exploring the immune response across a wide range of pathological contexts.

The minimal overlap between CD4^+^ and CD8^+^ T cell TCRαβ repertoire suggests the existence of intrinsic features in TCR sequences that determine the lineage commitment of double-positive T cells during thymic selection. Our analysis identified significant biases in the selection of V/J genes and amino acid usage in the CDRs of the TCRs associated with CD4^+^ and CD8^+^ T cells, suggesting that the TRAV/TRBV usage and the composition of amino acid usage after V(D)J recombination predispose TCRs to interact with MHC class I or II molecules^48,50^. These findings provide evidence supporting the plausibility of classifying CD4^+^ or CD8^+^ TCRs solely based on V/J gene usage and TCRαβ amino acid sequence. Such classification could enable the rapid identification and annotation of TCR repertoires in single-cell and bulk datasets without the need for additional markers, facilitating large-scale immune landscape studies across diverse populations and disease conditions. Furthermore, understanding the intrinsic biases in V/J gene usage and amino acid composition that drive lineage commitment provides key insights into the mechanisms of antigen recognition and T cell development. This knowledge can improve our ability to predict TCR–pMHC interactions, enhance the discovery of functional and disease-associated TCRs, and guide the development of targeted immunotherapies and vaccines.

Public TCRs are generated through convergent recombination, recombinatorial bias, and thymic selection, with their frequency further amplified during peripheral selection, making them more prevalent in activated T cells^9,10^. In our analysis, we indeed observed a higher abundance of public TCRs in the memory T cell population compared to the naïve population and shared among HLA-shared individuals, highlighting the critical role of peripheral survival and expansion in increasing the frequency of public TCRs^54,56^. Interestingly, these TCRs are frequently associated with specific antigens presented by shared HLA alleles across individuals, enabling the identification of disease-associated public TCRs within our population-level dataset. The most abundant and clonally expanded public TCRs in our data are known to have antigen specificity for virus-derived epitopes, such as EBV, CMV, IAV, and SARS-CoV-2. Meanwhile, we also identified numerous public TCRs shared in solid tumors without known epitope specificity, which may imply their roles in recognizing common antigens in different tumors.

Although the currently available public TCR analyses focused on the same V/J gene usage and CDR3 amino acid sequence, recent studies suggest that amino acids varied at particular positions in CDR3 are interchangeable without loss of publicness and antigen specificity^17,68^. These findings broaden the scope of TCR clustering strategies, enabling the identification of additional TCRs with shared antigen specificity. Our results also highlight the importance of TRAV genes in determining the CD4^+^/CD8^+^ lineage commitment, as the same TCRβ chain paired with TCRα chains using the same TRAV genes exhibit a higher propensity to belong to the same CD4⁺ or CD8⁺ lineage. This observation mirrors the phenomenon of light chain coherence found in antibodies in memory B cells^76^, underscoring the significant role of V genes in the light and α chains of both BCRs and TCRs. These findings suggest the importance of incorporating TRAV gene usage into TCR clustering analyses to more effectively identify TCRs with similar functional states. In parallel, recent methods emphasized the importance of transcriptome profiles in uncovering the relationships between TCR sequences and T cell phenotypes, as well as in distinguishing antigen-specific T cells from bystanders^22–25^. Finally, from a biological perspective, incorporating HLA and disease information is essential for identifying antigen-specific or disease-associated TCRs, whereas most previous methods have clustered TCRs unsupervised from this information.

As outlined by Hudson et al.^63^, unsupervised TCR clustering methods rely on either ‘hand-crafted’ features, such as sequence distance or motif enrichment, or representation learning with deep neural networks that do not incorporate prior knowledge about the relevance of specific amino acid positions. TCR-DeepInsight developed here followed the latter approach to incorporate the above-mentioned features and enabled better representation learning, model scalability, and extensibility. We employed the pre-trained large language model BERT, which has demonstrated its effectiveness in representation learning for DNA, protein, and TCR sequences^64–67,77^. Considering a notable association between CDR1/2/3 regions and T cell fate revealed in our analyses, we used the amino acid sequences of CDR1/2/3 from both TCR α and β chains as model input. In parallel, we adopted scAtlasVAE, a variational autoencoders (VAE)-based model for large-scale scRNA-seq data integration, batch effect removal, and transfer learning^28^. TCR-DeepInsight offers distinct advantages by not only integrating large-scale single-cell transcriptome data but also incorporating contextual information, such as disease condition and HLA genotypes, capable of handling million-scale immune profiling datasets all at once. Notably, leveraging the reference dataset generated in this study, TCR-DeepInsight is capable of identifying cTrGx-TCRαβ clusters characterized by convergent TCR sequences and transcriptomic profiles, suggesting shared functional roles across individuals. By integrating HLA and disease information, TCR-DeepInsight significantly enhances the biological insights that can be derived from single-cell immune profiling data, thereby advancing our understanding of T cell biology and contributing to the development of precision immunotherapies.

There are several limitations in our study. From a data-driven standpoint, for certain underrepresented disease conditions, the identification of disease-associated cTrGx-TCRαβ clusters may be influenced by an insufficient number of individuals included in the dataset. Since the number of TCRαβs collected in this study (1.4 × 10^6^) is just a fraction of the theoretical estimate of TCR diversity that could be as high as 10^15^, increasing the size and diversity of the dataset and incorporating TCR assembled from scRNA-seq^78^ in the future may significantly enhance the robustness of our tool, particularly in the identification of functional and disease-associated TCRs. Notably, the TCR-DeepInsight computational framework enables the swift adaptation of newly generated datasets and facilitates the identification of cTrGx-TCRαβ clusters in novel disease conditions using our reference as a background for comparison, which also allows the continuous enhancement of the reference and the tool. As a technical limitation, the TCR-DeepInsight framework aggregates embeddings at the clonotype level, without explicitly modeling clonal expansion or the progression of differentiation states. Additionally, the current architecture concatenates GEX and TCR embeddings using a fixed-weight hyperparameter, which does not account for potential interdependencies between these modalities. Future development of an end-to-end model that integrates GEX and TCR representations more effectively could enable adaptive learning of hyperparameters. Moreover, incorporating clonal expansion, antigen specificity, and structural information of TCRαβ may further improve the model’s performance in identifying cTrGx-TCRαβ clusters. Finally, it is important to note that most of the TCRαβ clonotypes identified within the cTrGx-TCRαβ clusters lack known antigen specificities, and some reported antigen-specific TCRαβs are based on tetramer or dextramer sorting and may be subject to non-specific binding. Therefore, more precise experimental validation is indispensable for confirming their antigen recognition. For clonotypes with unknown antigen specificity, future efforts incorporating high-throughput antigen screening platforms (e.g., yeast-display or combinatorial peptide library screening) will be essential to elucidate T cell responses in diverse biological contexts.

In the past, TCR-based immunotherapy and diagnostics have been constrained by both experimental and computational limitations. With the rapid advancements in single-cell omics and artificial intelligence-based computational tools, the prospects for precise TCR-based immunotherapy and diagnostics are increasingly promising. We envision that our population-level scTCRαβ reference and the TCR-DeepInsight tool represent a step forward in advancing future precise TCR-based immunotherapy and diagnostics.

## Materials and methods

### Data collection and integration of large-scale scTCR immune profiling datasets

We included additional datasets using a previously described preprocessing pipeline of single-cell immune profiling datasets with a scRNA-seq library (GEX library) and a scTCR-seq library (TCR library)^27^, in addition to those collected in our previous CD8^+^ T cell atlas^28^. In brief, cellranger (version 6.1.2) was used to obtain the gene expression count matrix (by *cellranger count* command) and TCR contig annotations (by *cellranger vdj* command) using raw sequencing reads as input. We include high-confidence T cells (hcT cells) defined as T cells passing filtering criteria on the number of captured genes and the percentage of mitochondrial gene-derived counts, and with full-length TCR sequences with V/J genes annotation and CDR3 sequence in both α and β chains from our previous collection. Altogether, our collection yielded millions of high-confidence T cells from 1017 biological samples (Supplementary Table S1). We annotated each sample with the individual ID, disease condition, and tissue origin. We used arcasHLA (version 0.5.0, IMGT reference version 3.46.0) to extract the HLA genotype of each individual using the aligned BAM files of the GEX library. After merging biological samples from the same individual, we annotated each individual with genotypes of HLA-A, HLA-B, HLA-C, HLA-DPB1, HLA-DRB1, HLA-DQA1, and HLA-DQB1.

We defined a unique TCRαβ clonotype as a TCR with the same TRBV, CDR3β amino acid sequence, TRBJ, TRAV, CDR3α amino acid sequence, and TRAJ in each individual. Note that public TCRαβs would be counted multiple times for each individual.

### Curation of publicly available bulk TCR sequencing datasets

Bulk TCR datasets from NCBI were downloaded using the *prefetch* command and converted using the *fastq*-*dump* command with the Sequence Read Archive (SRA) toolkit (version 3.0.0), while datasets from immuneACCESS (https://clients.adaptivebiotech.com/immuneaccess) were downloaded and unzipped manually. After retrieving the raw FASTQ files, the quality assessment was conducted with FastQC (version 0.11.9). Quality control and adapter trimming were performed using Trimmomatic^79^ (version 0.3.9) with the following settings: ILLUMINACLIP (2:30:10) for adapter removal, SLIDINGWINDOW (8:25) for quality trimming, LEADING/TRAILING (25) for removing low-quality bases, while single-end (SE) or paired-end (PE) mode was selected according to the samples.

After the quality control step, alignment and assembly were conducted via MIXCR^80^ (version 4.4.2) with the preset *generic-tcr-amplicon* command. Since the input data was DNA-based, the --dna parameter was specified. To accurately reconstruct the TCR repertoire, the --floating-left-alignment-boundary parameter allowed flexible alignment of the V segment’s left boundary, while *the --rigid-right-alignment-boundary* parameter ensured a fixed alignment of the J segment’s right boundary. Additionally, the *--keep-non-CDR3-alignments* parameter was enabled to retain alignments without identifiable CDR3 regions, ensuring a comprehensive analysis. The *--species* parameter was set to human (Homo sapiens). The choice of SE or PE modes was also considered. Other parameters were default.

After a further round of data cleaning, the processed human bulk TCR datasets contain a total of 94,910,428 full-length TCRβ sequences containing TRBV, CDR3β, and TRBJ from 125 projects. Metadata of these projects is extracted from the TCRdb^31^ and immuneACCESS, covering 63 types of disease and healthy samples, and 7,738,172 TCRβ sequences from sorted CD8^+^ T cells, 6,263,093 from CD4^+^ T cells, 340,834 CD4^+^ Treg, and 22,176 from MAIT cells.

### Building a reference of TCRαβs with antigen specificity

We collected datasets with TCR–pMHC binding pairs from experimental data. We included datasets using pMHC-tetramer cell sorting together with single-cell paired TCRα/β amplification. Paired TCRα/β with CDR3 amino acid sequences were obtained from curated databases, including McPAS-TCR^81^, VDJdb^82^, and TCRdb^31^. We also include recently released datasets using combined DNA-barcoded pMHC tetramer/dextramer and single-cell RNA-sequencing from 10x genomics (https://www.10xgenomics.com/cn/resources/datasets/cd-8-plus-t-cells-of-healthy-donor-1-1-standard-3-0-2, https://www.10xgenomics.com/cn/resources/datasets/cd-8-plus-t-cells-of-healthy-donor-2-1-standard-3-0-2, https://www.10xgenomics.com/cn/resources/datasets/cd-8-plus-t-cells-of-healthy-donor-3-1-standard-3-0-2, https://www.10xgenomics.com/cn/resources/datasets/cd-8-plus-t-cells-of-healthy-donor-4-1-standard-3-0-2) and recent publications^58,70^. After removing repetitive data records and data curation, we obtained 28,223 TCR-pMHC pairs with paired CDR3α and CDR3β amino acid sequences and 828 peptide epitopes.

### Training a VAE model for the universal representation of transcriptome features

We integrated the transcriptome features of the hcT cells, including both CD4^+^ and CD8^+^ T cells, by learning the batch-corrected latent embedding with scAtlasVAE (version 1.0.4)^28^ using 3000 highly variable genes (HVGs) with default parameters.

We annotated the hcT cells with CD4^+^ or CD8^+^ lineage by gene count of *CD4*, *CD8A*, and *CD8B*, followed by the nearest neighbor classifier (n_neighbors = 13) on the latent embedding to categorize cells with double-positive or double-negative *CD4* or *CD8* expression into CD4^+^ or CD8^+^ T cells^28^. The CD4^+^ T cells were further categorized into CD4^+^ naïve T cells, CD4^+^ memory T cells, CD4^+^ T regulatory cells, CD4^+^ follicular helper T cells, CD4^+^*CD40LG*^+^ T cells and CD4^+^ cycling T cells by key marker genes including *SELL* (Selenoprotein L), *TCF7* (Transcription Factor 7), *FOXP3* (Forkhead Box P3), *CXCR5* (CXC motif chemokine receptor 5), *CD40LG* (CD40 Ligand*)*, *IFNG* (Interferon-gamma), and *MKI67* (Marker Of Proliferation Ki-67). The CD8^+^ T cells were further divided into CD8^+^ naïve T cells, CD8^+^ memory T cells, CD8^+^ recently activated effector memory T cells, CD8^+^ exhausted T cells, CD8^+^ effector T cells, CD8^+^ cycling T cells, CD8^+^ ILTCK-LC), and CD8^+^ MAIT/iNKT cells, based on key marker genes including *SELL*, *TCF7*, *CXCL13* (C-X-C Motif Chemokine Ligand 13), *PDCD1* (Programmed Cell Death 1), *KLRB1* (Killer Cell Lectin Like Receptor B1), and *ZBTB16* (Zinc Finger And BTB Domain Containing 16).

We merged the T cells with the same α and β chains defined by TRAV-CDR3α-TRAJ and TRBV-CDR3β-TRBJ in each individual and obtained unique TCRαβ clonotypes. Each unique TCRαβ was annotated with T cell subtypes by their deriving cells, where the conventional CD4^+^ T_conv_ includes CD4^+^ T_n_, CD4^+^ T_m_, CD4^+^ TFH, and CD4^+^ cycling T cells. The transcriptome feature of each T cell or clonotype was visualized by projecting the learned latent embedding into a 2-dimensional space using the UMAP algorithm^83^.

### Identifying enriched V/J genes in the T cell subtype

Odds ratio and *P* value were calculated using Fisher’s exact test to discover T cell subtype-specific V/J joining in α and β chains and TRAV-TRBV pairing. The odds ratio of a given V/J combination (${{\rm{C}}}_{{\rm{VJ}}}$) in a set of T cells of the same subtype (${{\rm{T}}}_{{\rm{type}}}$) against all other T cells (${{\rm{T}}}_{{\rm{others}}}$) is given by:

1 $${\rm{OR}}=\frac{\left|{{\rm{C}}}_{{\rm{VJ}}}^{+}\in {{\rm{T}}}_{{\rm{type}}}\right|\times \left|{{\rm{C}}}_{{\rm{VJ}}}^{-}\in {{\rm{T}}}_{{\rm{others}}}\right|}{\left|{{\rm{C}}}_{{\rm{VJ}}}^{-}\in {{\rm{T}}}_{{\rm{type}}}\right|\times \left|{{\rm{C}}}_{{\rm{VJ}}}^{+}\in {{\rm{T}}}_{{\rm{others}}}\right|}$$

The calculation of *P* value followed by multiple testing corrections with the Bonferroni method was achieved by the SciPy Python package (version 1.10.0)^84^. We reported V/J combinations preferentially selected by certain T cell types by thresholding the adjusted *P* value < 0.05, odds ratio > 2, and the number of supporting cells greater than 400. The selected cell type enriched V/J combinations are visualized by a Sankey plot using the number of cells on the edges of each V/J combination.

### Analysis of amino acid usage in the middle region of the CDR3 sequence

The middle region of the CDR3 sequence in α or β chain (CDR3α-mr and CDR3β-mr) was defined as the amino acid encoded by random nucleotide insertions between the V and J segments. After removing the amino acids derived from the V and J segments of the CDR3 sequence, the remaining amino acids were annotated with the CDR3-mr sequence. The percentage of amino acid usage grouped by their chemical properties was calculated for each TCR, and then averaged across different T cell subtypes (CD8^+^ T, CD4^+^ T_conv,_ and CD4^+^ T_reg_). The significance of the difference in the percentage between different T cell subtypes was calculated by paired-sample two-tailed Student’s *t*-test for scTCR-seq, and two-tailed Student’s *t*-test for bulk TCR-seq.

### Definition and analysis of public TCRs

Public TCRs were defined independently by either the single α or β chain or paired α/β chains. Public TCRαβs were TCRs that occur in at least two individuals who share the exact same TRBV, CDR3β amino acid sequence, TRBJ, TRAV, CDR3α amino acid sequence, and TRAJ, while public TCRα and TCRβ only consider the same V/J gene and CDR3 amino acid sequence of a single α or β chain. The V and J segments adopted the annotation from the output of cellranger.

We calculated the generation probability of α and β chains for each public TCR and non-public TCR by OLGA (version 1.2.4)^51^. The generation probability models for the α and β chains are initialized by the *GenerationProbabilityVJ* and *GenerationProbabilityVDJ* functions, which accept both the CDR3 amino acid sequence and V/J gene as input. The generation probability was followed by a negative log-transformation to get the logP_gen_ of each TCR. The significance of the difference in logP_gen_ between public and non-public TCRs was calculated by a paired two-tailed Student’s *t*-test.

### Association analysis between public TCRs, HLA genotype, and T cell phenotype

Public TCRs were classified into two groups: those with at least one shared MHC class I or class II HLA allele, and those without any shared alleles. Concurrently, public TCRs were further classified as CD8^+^ or CD4^+^ based on the predominant T cell type among cells expressing the respective public TCR. The odds ratio of CD8^+^ against CD4^+^ T cells in public TCRs with or without shared class I HLA allele is given by:

2 $${\mathrm{OR}}_{{\mathrm{CD}8}^{+}|\mathrm{class\; I}}=\frac{\left|{\mathrm{Pub}}_{{\mathrm{CD}8}^{+}}^{\mathrm{class}\,{{\rm{I}}}^{+}}\right|\times \left|{\mathrm{Pub}}_{{\mathrm{CD}4}^{+}}^{{\mathrm{class\; I}}^{-}}\right|}{\left|{\mathrm{Pub}}_{{\mathrm{CD}8}^{+}}^{{\mathrm{class\; I}}^{-}}\right|\times \left|{\mathrm{Pub}}_{{\mathrm{CD}4}^{+}}^{{\mathrm{class\; I}}^{+}}\right|}$$

Where ${{\rm{Pub}}}_{{{\rm{CD}}8}^{+}}^{{{\rm{class\; I}}}^{+}}$ denotes public TCRs from class I MHC-shared individuals with CD8^+^ as the dominant cell type. Similarly, the odds ratio of CD8^+^ against CD4^+^ T cells in public TCRs with or without shared class II HLA allele is given by:

3 $${{\rm{OR}}}_{{{\rm{CD}}4}^{+}|{\rm{class\; II}}}=\frac{\left|{{\rm{Pub}}}_{{{\rm{CD}}4}^{+}}^{{{\rm{class\; II}}}^{+}}\right|\times \left|{{\rm{Pub}}}_{{{\rm{CD}}8}^{+}}^{{{\rm{class\; II}}}^{-}}\right|}{\left|{{\rm{Pub}}}_{{{\rm{CD}}4}^{+}}^{{{\rm{class\; II}}}^{-}}\right|\times \left|{{\rm{Pub}}}_{{{\rm{CD}}8}^{+}}^{{{\rm{class\; II}}}^{+}}\right|}$$

The significance of the odds is given by Fisher’s exact test.

### Training BERT model for the universal representation of TCRαβ sequences

We adopted the BERT model, originally developed in the field of natural language processing, to obtain a scalable representation of the TCRαβ sequence. The transformer-based models were shown to outperform conventional encoding models, including multilayer perceptron (MLP), convolutional networks, and recurrent models, using attention mechanisms to capture inter-relationships between tokens. The BERT model was implemented in Python using the PyTorch framework and Hugging Face’s Transformer libraries, with hyperparameters where hidden dimensionality = 192, intermediate size = 768, number of attention heads = 6, and number of hidden layers = 6. We implemented a tokenizer combining both CDR1α, CDR2α, CDR3α, CDR1β, CDR2β, and CDR3β amino acid sequence, adding a classification token (CLS, ‘^’) ahead of CDR1α, gap tokens (GAP, ‘:’) between the TCRα and TCR β chains, and padding tokens (PAD, ‘.’) to align CDR3α and CDR3β sequence to the same encoding length. The length of the tokenized sequence contains 110 tokens, for TCRαβs with CDR3α and CDR3β within 36 amino acids.

For example, a T cell receptor comprising the TRAV1-2 and TRAJ20 gene segments with the CDR3α sequence CVWGLDYKLSF, and the TRBV10-2 and TRBJ2-3 gene segments with the CDR3β sequence CASARLVGADTQYF, would be represented as follows:

TSGFNG:NVLDGL:CVWGLDYKLSF:WSHSY:SAAADI:CASARLVGADTQYF

by matching V genes to CDR1 and CDR2 amino acid sequences, and then

^WSHSY…:SAAADI..:CASARLVGADTQYF………………….:TSGFNG..:NVLDGL..:CVWGLDYKLSF…………………….

followed by inserting CLS, GAP, and PAD tokens.

The embeddings of the token from the CDR1α, CDR2α, CDR3α, CDR1β, CDR2β, and CDR3β amino acid sequences were used to represent the whole TCRαβ after a mean pooling operation. The 192-dimensional embedding BERT output could be projected into a 50-dimensional space via PCA by using the implementation of scikit-learn. The 50-dimensional representation of the TCR was then called the TCR embedding.

### Unsupervised TCRαβ clustering with shared transcriptome state

We aggregated the GEX embedding from the VAE model for each unique TCRαβ clonotype. We concatenated the TCR embeddings and the aggregated GEX embeddings and obtained a TCR-GEX joint representation of TCRαβ clonotypes. We adopted faiss-gpu (version 1.7.2), a computational framework for rapid similarity search optimized and accelerated by GPU^85^, for indexed *k*-nearest neighbor search of TCR-GEX joint representation based on Euclidean distance.

We defined the distance between two TCRαβs ${{\boldsymbol{s}}}_{{\boldsymbol{i}}}$ and ${{\boldsymbol{s}}}_{{\boldsymbol{j}}}$ in our TCR-GEX joint representation space as TrGx distance ($\text{TrG}{\text{x}}_{\text{Distance}}$), which is the Euclidean distance between the TCR-GEX joint representation of TCRαβs.

4 $${{\text{TrGx}}}_{\text{Distance}}\left({s}_{i},{s}_{j}\right)={\text{EuclideanDistance}}\left({\text{TrGx}}\left({s}_{i}\right),{\text{TrGx}}\left({s}_{j}\right)\right)$$

We use the following strategy to cluster HLA-shared cTrGx-TCRαβs. First, each TCRαβ clonotype in our datasets was used as an anchor to find the *k*-nearest neighbors (*k* = 100 by default). We then select the top $n$ TCRαβ clonotypes ranked by the $\text{TrG}{\text{x}}_{\text{Distance}}$ with at least one shared HLA allele as a cTrGx-TCRαβ cluster.

The strategy for disease-associated cTrGx-TCRαβs is similar to the one above, while ranking the TCRαβ clonotype by $\text{TrG}{\text{x}}_{\text{Distance}}$ with the same disease type, and grouping the anchor TCRαβ and the neighbor TCRαβ clonotypes as a potential disease-associated cluster. In the following neighbor search, we removed the possibility of the neighbor TCRαβ to prevent repeated neighbor TCRαβs clusters.

We defined the TrGx convergence score ($\text{TrG}{\text{x}}_{\text{Convergence}}$) to measure the sequence similarity of TCRαβ clonotypes within a cTrGx-TCRαβ cluster. The similarity score was defined as the mean Euclidean distance in the TCR-GEX joint representation space between each neighboring TCRαβ clonotype (${s}_{i}$) inside the cluster and the anchor TCRαβ clonotype (${{\boldsymbol{s}}}_{\text{anchor}}$). A negative transformation was then applied so that higher scores indicate stronger convergence:

5 $${{\text{TrGx}}}_{{\text{Convergence}}}=-\frac{{\sum }_{i=1}^{n}{{\text{TrGx}}}_{{\text{Distance}}}\left({s}_{i},{s}_{{\text{anchor}}}\right)}{n}$$

where $n$ is the number of neighbor TCRs in the cluster.

We defined a TrGx disease-association score ($\text{TrG}{\text{x}}_{\text{Disease}-\text{association}}$) to measure the distinctiveness of TCRαβ sequence and gene expression profile in a disease-associated cluster. Specifically, the score was calculated by the Euclidean distance between all neighboring TCRαβ in the cluster and the top-ranked TCRs that are most similar to the anchor TCRαβ, but derived from different diseases. The latter was determined with an equal number of neighbors of the anchor TCRαβ.

6 $${{\text{TrGx}}}_{{\text{Disease}}-{\text{association}}}=\frac{{\sum }_{j=1}^{m}{{\text{TrGx}}}_{{\text{Distance}}}\left({s}_{j},{s}_{{\text{anchor}}}\right)-{\sum }_{i=1}^{n}{{\text{TrGx}}}_{{\text{Distance}}}\left({s}_{i},{s}_{{\text{anchor}}}\right)}{n+1}$$

where $m$ equals to $n+1$, which is the number of unique TCRαβ clonotypes in a cTrGx cluster, and ${s}_{j}$ indicates TCRαβ clonotype in the background repertoire. The proposed score enables a more accurate evaluation of the uniqueness of TCRαβ in a disease-associated cluster.

### Selecting disease-associated cTrGx-TCRαβ clusters

The selection of disease-associated cTrGx-TCRαβ clusters involved the use of previously established $\text{TrG}{\text{x}}_{\text{Convergence}}$ and $\text{TrG}{\text{x}}_{\text{Disease}-\text{association}}$. Specifically, cTrGx-TCRαβ clusters with $\text{TrG}{\text{x}}_{\text{Disease}-\text{association}}$ greater than a user-determined threshold is selected for further analysis ($\text{TrG}{\text{x}}_{\text{Disease}-\text{association}} > 2$ in this study). Additional criteria are applied based on the number of unique individuals (more than 1 in this study) and unique TCRαβ (more than 2 in this study) in a cTrGx-TCRαβ cluster.

To assess the statistical significance of the cTrGx-TCRαβ clusters to be distinct from the background repertoire, we used a permutation-based test. Under the null hypothesis that the observed $\text{TrG}{\text{x}}_{\text{Disease}-\text{association}}$ could arise by chance, we randomly permuted TCRαβ assignments across cells while keeping HLA and disease labels fixed, and recalculated the $\text{TrG}{\text{x}}_{\text{Disease}-\text{association}}$ for each permutation. The *P* value was computed as the fraction of permuted scores that were equal to or greater than the observed score, since a smaller $\text{TrG}{\text{x}}_{\text{Disease}-\text{association}}$ indicates distinct communities. Throughout this study, we reported cTrGx-TCRαβ clusters with permutation *P* values less than 0.05.

### Comparison between TCR-DeepInsight and other methods

We compare the clustering result from TCR-DeepInsight with methods including:

GLIPH2^16^. We used GLIPH2 (available at http://50.255.35.37:8080/tools) with default parameters. GLIPH2 takes CDR3β, TRBV, TRBJ, and CDR3α as model input.

GIANA^19^. We used GIANA (available at https://github.com/s175573/GIANA) with default parameters. GIANA takes CDR3β and TRBV as model input.

clusTCR^21^. We use the clustcr Python package (version 1.0.2) with default parameters. Clustcr takes CDR3β and CDR3α as model input.

iSMART^20^. We use iSMART (available at https://github.com/s175573/iSMART) with default parameters. iSMART takes CDR3β and TRBV as model input.

The entropy of TRBV, TRAV, and cell type usage in each cluster is defined as

7 $${\rm{Entropy}}\left({\rm{D}}\right)=-\sum _{{\rm{d}}\in {\rm{D}}}{\rm{p}}\left({\rm{d}}\right){\log }_{{\rm{e}}}\left({\rm{p}}\left({\rm{d}}\right)\right)$$

Where ${\rm{D}}$ is the set of TRBV, TRAV, or cell type in each cluster.

We compare the joint representation from TCR-DeepInsight with methods including:

scNAT^24^. We use the scNAT-biqing-zhu Python package (version 0.0.1). scNAT takes CDR3β, TRBV, TRBJ, and GEX with the same 3000 HVGs as used in our study as model input.

mvTCR^25^. We use the mvTCR Python package (version 0.2.1.1) with default parameters. mvTCR takes CDR3β, CDR3α, and GEX with the same 3000 HVGs as used in our study as model input.

MIST^26^. We use the MIST Python package (version 1.0.0) with default parameters. MIST takes CDR3β, TRBV, TRBJ, CDR3α, TRAV, TRAJ, and GEX with the same 3000 HVGs as used in our study as model input.

### Querying new paired scRNA-seq and scTCR-seq samples against existing reference

To transfer the transcriptome latent embedding, we used the data transfer functionality from the scAtlasVAE model. Specifically, during data transfer, the weights of the encoder and decoder were kept the same as the VAE model before transfer learning, and the original set of batch indexes would be extended to allow for extended datasets. The pre-trained BERT model is used for obtaining embeddings from the query TCR sequences.

We used a recently published scRNA-seq and scTCR-seq derived from AS^71^ and melanoma patients^72^. We kept the same 3000 HVGs as in our previous analysis to perform transfer learning. We then aggregated the transferred GEX embedding by unique TCRs and concatenated it to the TCR embedding from the BERT model, followed by PCA transformation with the same weight as the reference data. Categorizing AS-associated cTrGx-TCRαβ clusters followed the same strategy described above, and TCRαβ clusters with more than 1 unique individual and more than 2 unique TCRαβs were selected.

## Supplementary information

Supplementary Figures

Supplementary Table S1

Supplementary Table S2

Supplementary Table S3

Supplementary Table S4

Supplementary Table S5

Supplementary Table S6

Supplementary Table S7

## Data Availability

The constructed single-cell TCR immune profiling reference built and analyzed in this study (excluding the two datasets with controlled access) and bulk TCR sequencing reference datasets can be accessed at Zenodo (https://zenodo.org/records/12741480).

## Code availability

The TCR-DeepInsight method is available as an open-source Python package at https://github.com/WanluLiuLab/TCR-DeepInsight with additional documentation available at https://tcr-deepinsight.readthedocs.io/en/latest/. A Jupyter Notebook including codes and raw output figures is available at https://huarc.net/notebook/tcr-deep-insight/index.html.
