# Supplementary material for: A pan-disease and population-level single-cell TCRαβ repertoire reference
Source: Cell Discov. 2025 Oct 14;11:82. doi: 10.1038/s41421-025-00836-7 (PMC12521495; doi:10.1038/s41421-025-00836-7)
Supplement: Supplementary file 1 — Supplementary Figures [file 41421_2025_836_MOESM1_ESM.pdf]

SUPPLEMENTARY FIGURES & LEGENDS
A pan-disease and population-level single-cell TCRαβ repertoire reference

Correspondence: lu_linrong@zju.edu.cn and wanluliu@intl.zju.edu.cn

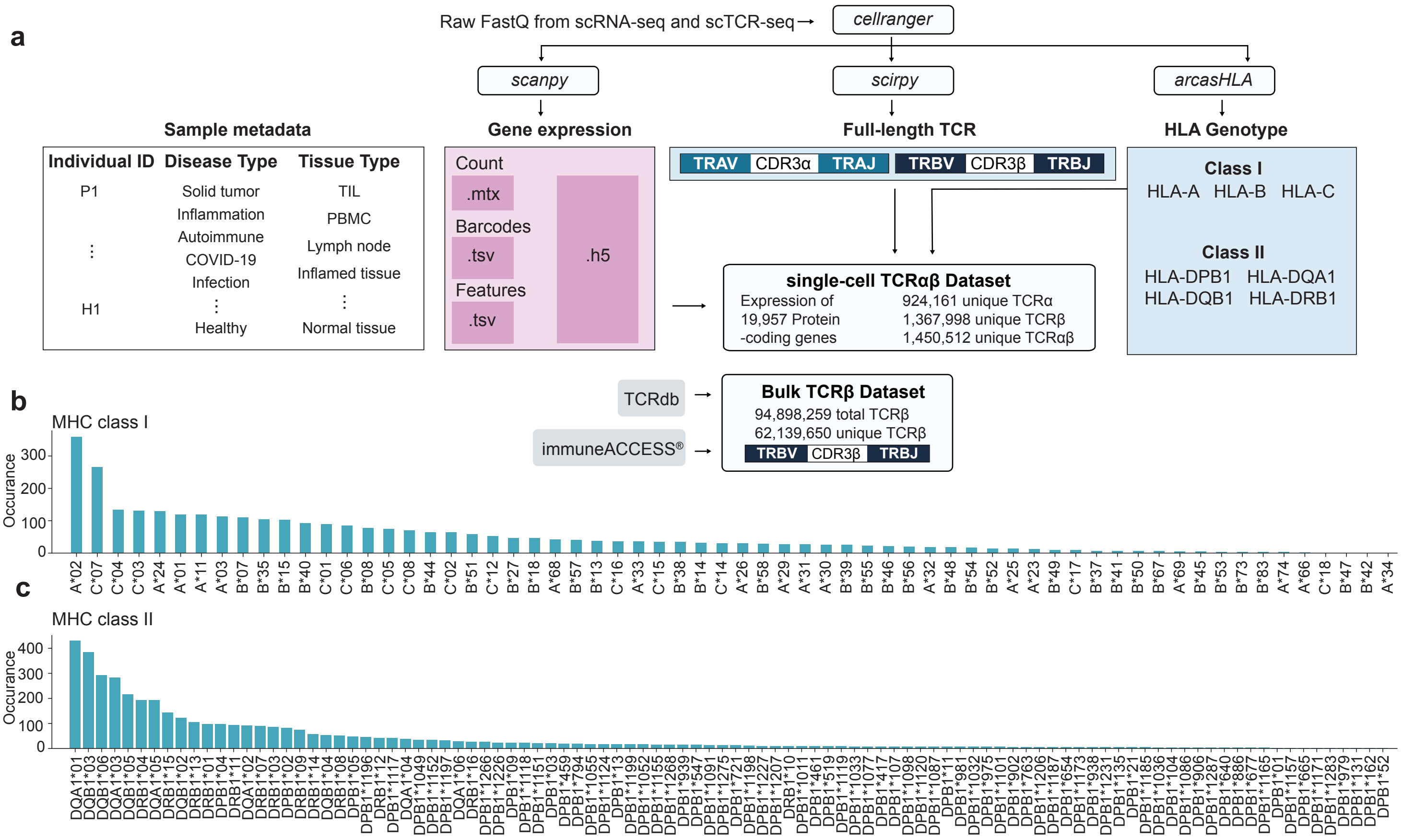

Supplementary Figure 1 | Overview of the pan-disease single-cell TCRαβ repertoire reference atlas. a, Workflow illustrating the standardized preprocessing steps and data composition of the pan-disease TCRαβ repertoire reference atlas. Raw scRNA-seq and scTCR-seq data were processed using Cell Ranger, followed by Scanpy and Scirpy for analysis. Alignment BAM files were analyzed with arcasHLA for HLA genotype inference. Data acquisition and summary of the bulk TCRβ sequencing dataset. **b**,**c**, Bar plots displaying the frequency distribution of MHC class I (**b**) and MHC class II (**c**) HLA alleles.

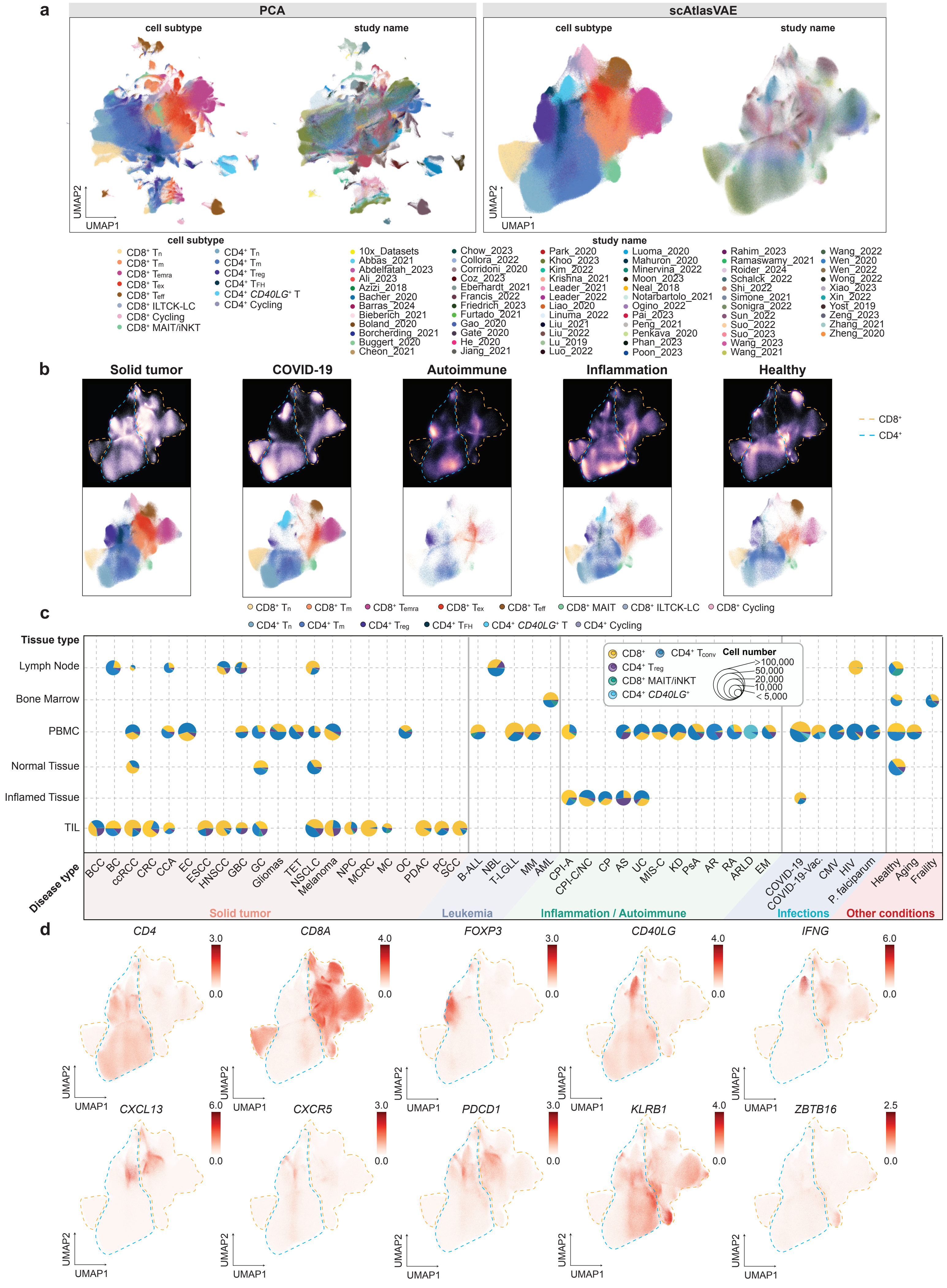

a
PCA
scAtlasVAE
cell subtype
study name
UMAP2
UMAP1
CD8⁺ Tn
CD8⁺ Tm
CD8⁺ Temra
CD8⁺ Tex
CD8⁺ Teff
CD8⁺ ILTCK-LC
CD8⁺ Cycling
CD8⁺ MAIT/iNKT
CD4⁺ Tn
CD4⁺ Tm
CD4⁺ Treg
CD4⁺ TFH
CD4⁺ CD40LG⁺ T
CD4⁺ Cycling
10x_Datasets
Abbas_2021
Abdelfatah_2023
Ali_2023
Azizi_2018
Bacher_2020
Barras_2024
Bieberich_2021
Boland_2020
Borcherding_2021
Buggert_2020
Cheon_2021
Chow_2023
Collora_2022
Corridoni_2020
Coz_2023
Eberhardt_2021
Francis_2022
Friedrich_2023
Furtado_2021
Gao_2020
Gate_2020
He_2020
Jiang_2021
Park_2020
Khoo_2023
Kim_2022
Krishna_2021
Leader_2021
Leader_2022
Liao_2020
Linuma_2022
Liu_2021
Liu_2022
Lu_2019
Luo_2022
Luoma_2020
Mahuron_2020
Minervina_2022
Moon_2023
Neal_2018
Notarbartolo_2021
Ogino_2022
Pai_2023
Peng_2021
Penkava_2020
Phan_2023
Poon_2023
Rahim_2023
Ramaswamy_2021
Roider_2024
Schalck_2022
Shi_2022
Simone_2021
Sonigra_2022
Sun_2022
Suo_2022
Suo_2023
Wang_2023
Wang_2021
Wang_2022
Wen_2020
Wen_2022
Wong_2022
Xiao_2023
Xin_2022
Yost_2019
Zeng_2023
Zhang_2021
Zheng_2020
b
Solid tumor
COVID-19
Autoimmune
Inflammation
Healthy
CD8⁺
CD4⁺
c
CD8⁺ Tn
CD8⁺ Tm
CD8⁺ Temra
CD8⁺ Tex
CD8⁺ Teff
CD8⁺ MAIT
CD8⁺ ILTCK-LC
CD8⁺ Cycling
CD4⁺ Tn
CD4⁺ Tm
CD4⁺ Treg
CD4⁺ TFH
CD4⁺ CD40LG⁺ T
CD4⁺ Cycling
Tissue type
Lymph Node
Bone Marrow
PBMC
Normal Tissue
Inflamed Tissue
TIL
CD8⁺
CD4⁺ Tconv
CD4⁺ Treg
CD8⁺ MAIT/iNKT
CD4⁺ CD40LG⁺
Cell number
>100,000
50,000
20,000
10,000
< 5,000
Disease type
BCC
BC
ccRCC
CRC
CCA
EC
ESCC
HNSCC
GBC
GC
Gliomas
TET
NSCLC
Melanoma
NPC
MCRC
MC
OC
PDAC
PC
SCC
B-ALL
NBL
T-LGLL
MM
AML
CPI-A
CPI-C/NC
CP
AS
UC
MIS-C
KD
PsA
AR
RA
ARLD
EM
COVID-19
COVID-19-Vac.
CMV
HIV
P. falciparum
Healthy
Aging
Frailty
Solid tumor
Leukemia
Inflammation / Autoimmune
Infections
Other conditions
d
CD4
CD8A
FOXP3
CD40LG
IFNG
CXCL13
CXCR5
PDCD1
KLRB1
ZBTB16
3.0
4.0
6.0
2.5
0.0
UMAP2
UMAP1

Supplementary Figure 2 | Integration of the $CD4^{+}$ and $CD8^{+}$ T cells by GEX features.
a, Uniform Manifold Approximation and Projection (UMAP) of the integrated datasets colored by T cell subtypes and batch sources (study names) using PCA (left) or scAtlasVAE embeddings (right). **b**, UMAP view of cell density (top) and states (bottom) displaying T cell distribution across 5 major disease types. **c**, Pie charts summarizing T cell types categorized by tissue type and disease type. **d**, UMAP plots of the normalized expression of key marker genes to define T cell types.

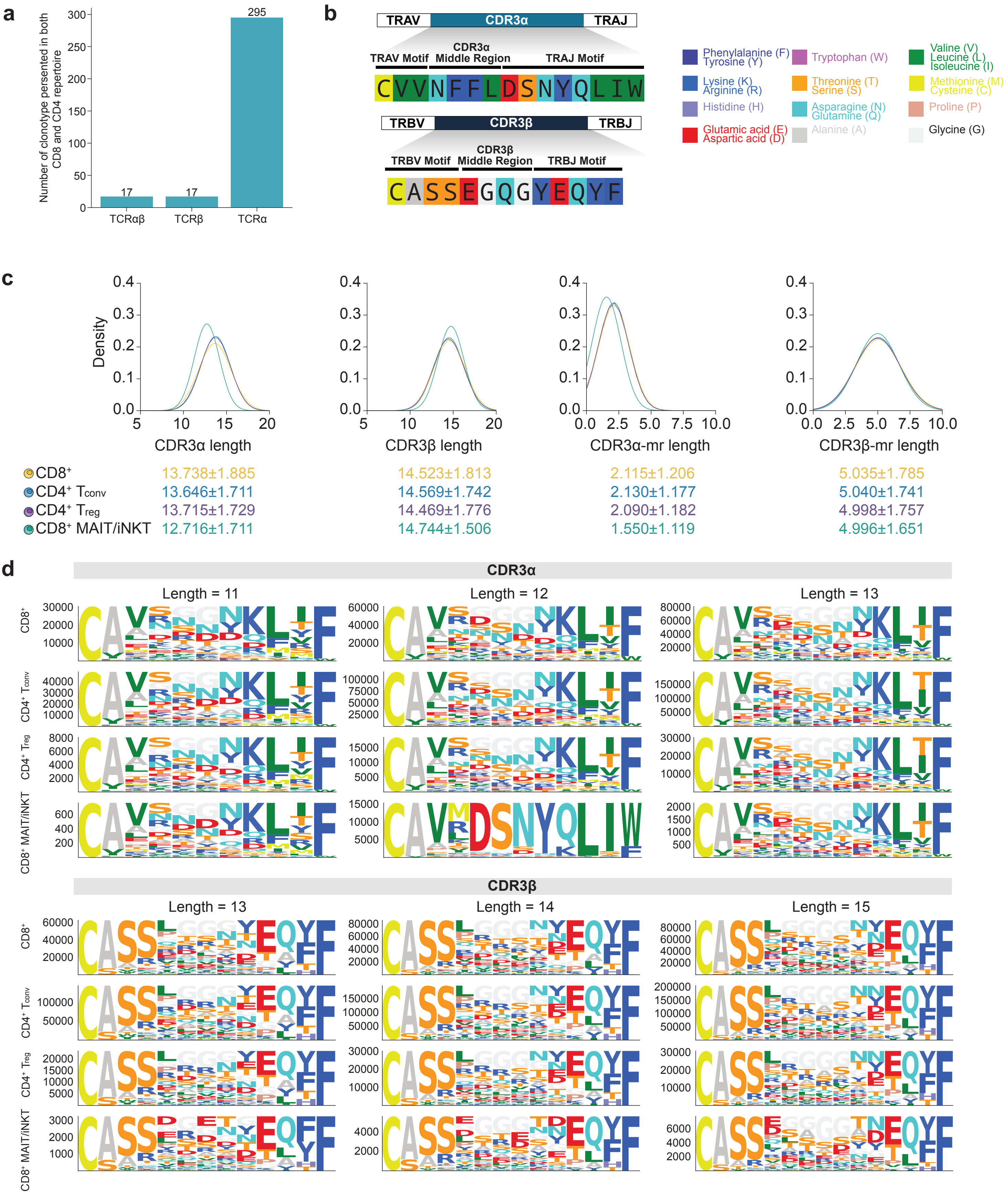

Supplementary Figure 3 | Length characteristics of TCRαβs and amino acid usage of the CDR3 region of major T cell subtypes. a, Bar plot of TCRαβ, TCRβ, and TCRα clonotypes overlapped in $CD8^+$ and $CD4^+$ repertoire. $CD8^+$ repertoire is defined by TCRαβs from T cells with gene expression count *CD8A/CD8B* > 0 and CD4 = 0, and $CD4^+$ repertoire is from T cells with *CD4* > 0 and *CD8A&CD8B* = 0. Overlapping clonotypes are identified as those present in both $CD8^+$ and $CD4^+$ repertoires, with a total cell count exceeding 20 and a ratio of $CD8^+$ to $CD4^+$ T cells between 0.5 and 2. **b**, Schematic diagram of the CDR3 middle region of a TCR. **c**, Length distribution of the CDR3 and CDR3 middle region amino acid sequence of major T cell subtypes. **d**, Motif logo plots for CDR3α and CDR3β, grouped by sequence length of major T cell subtypes.

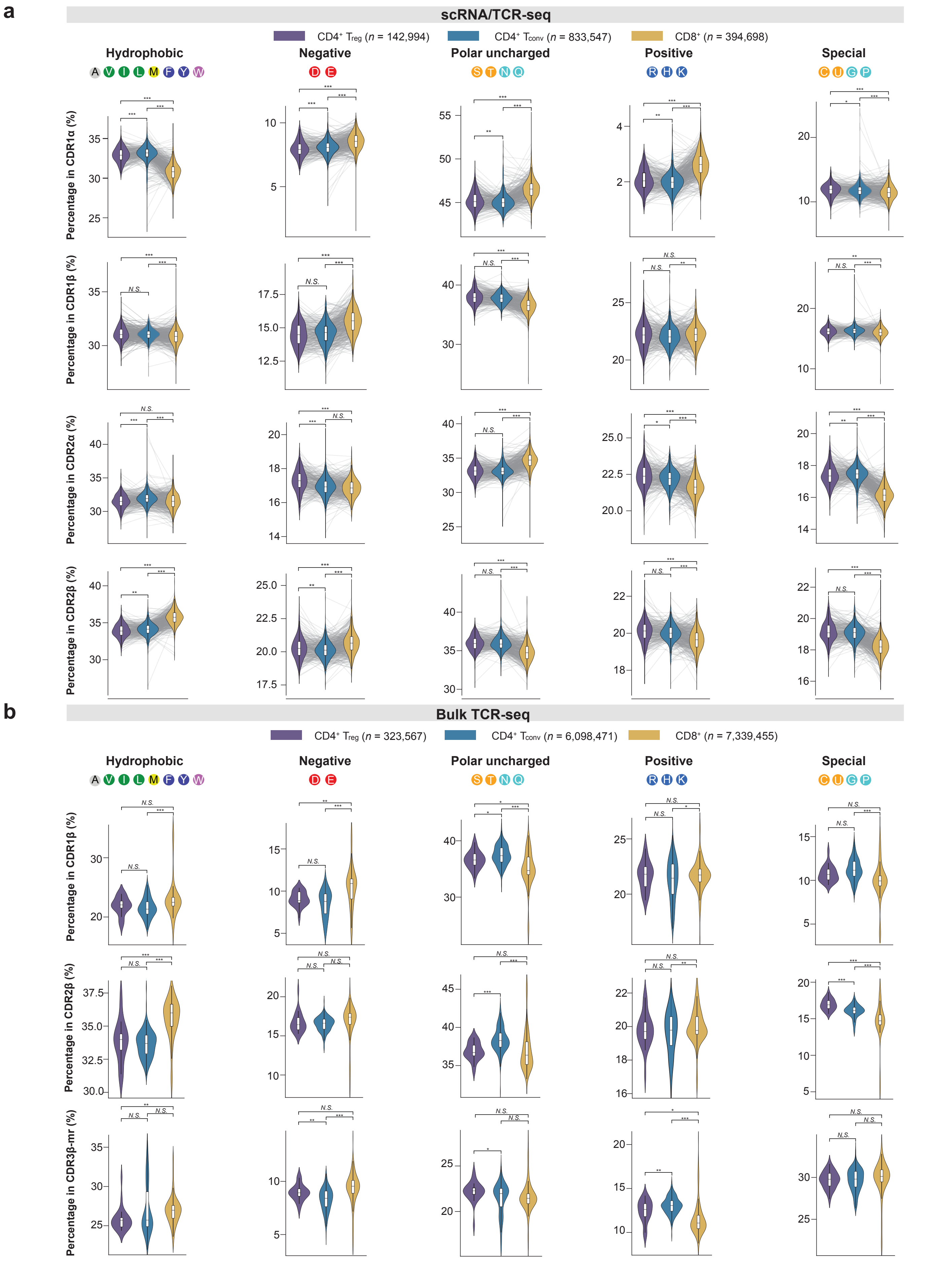

Supplementary Figure 4 | Amino acid properties of the CDR1 and CDR2 region from single-cell and bulk TCR-seq. a,**b**, Violin plot showing the preference for amino acids with different physicochemical properties in CDR1 and CDR2 for TCRα and TCRβ chains from scRNA/TCR-seq (**a**) and CDR1 and CDR2 TCRβ chains from bulk TCR-seq (**b**) of CD4 T_{reg}, CD4 T_{conv}, and CD8 cells. For scRNA/TCR-seq, each gray dot in the plot represents data from one individual, while lines connecting gray dots indicate amino acid usage differences in different cell types within each individual, considering support from at least 100 cells. White dots within the violin plot represent the average percentage of amino acid usage. Letters within colored dots represent the various physico-chemical properties of amino acids. *** *P*-value < 0.001, ** *P*-value < 0.01, * *P*-value < 0.05, paired *t*-test for scRNA/TCR-seq and *t*-test for bulk TCR-seq; *N.S.* not significant.

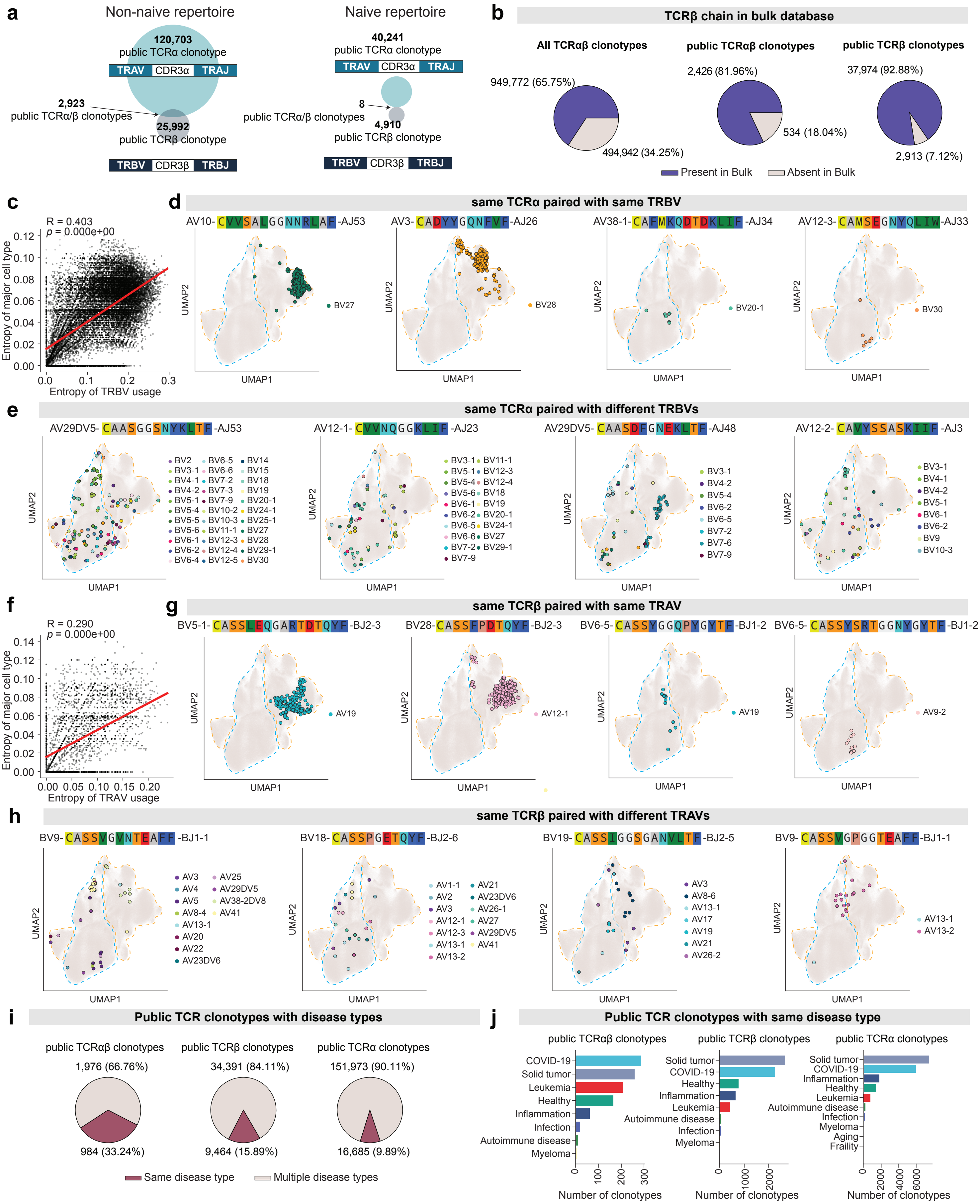

Supplementary Figure 5 | Additional characteristics of public TCRs. a, Public TCRαβs in naïve and non-naïve single-cell TCRαβ repertoire. **b**, Pie chart depicting the distribution of TCRβ chains from public TCRαβ and TCRβ clonotypes identified within the bulk dataset. **c**,**f**, Scatter plots of Shannon entropy of TRBV usage and Shannon entropy of major cell type in the same TCRα paired with different TRBVs (**c**) and same TCRβ paired with different TRAVs (**f**). Red lines indicate Pearson correlation fits; correlation coefficients (R) and associated *p*-values are reported. **d**,**e**, UMAP projections of representative examples of TCRαs paired with the same (**d**) or different TRBVs (**e**). **g**,**h**, UMAP projections of representative examples of TCRβs paired with the same (**g**) or different TRAVs (**h**). Each point denotes a single cell, colored by the corresponding V gene used. **i**, Distribution of public TCRαβ, TCRβ, and TCRα clonotypes, each associated with distinct disease categories, represented in pie chart format. **j**, Bar plots of the number of public TCRαβ, TCRβ, and TCRα clonotypes categorized by specific disease types.

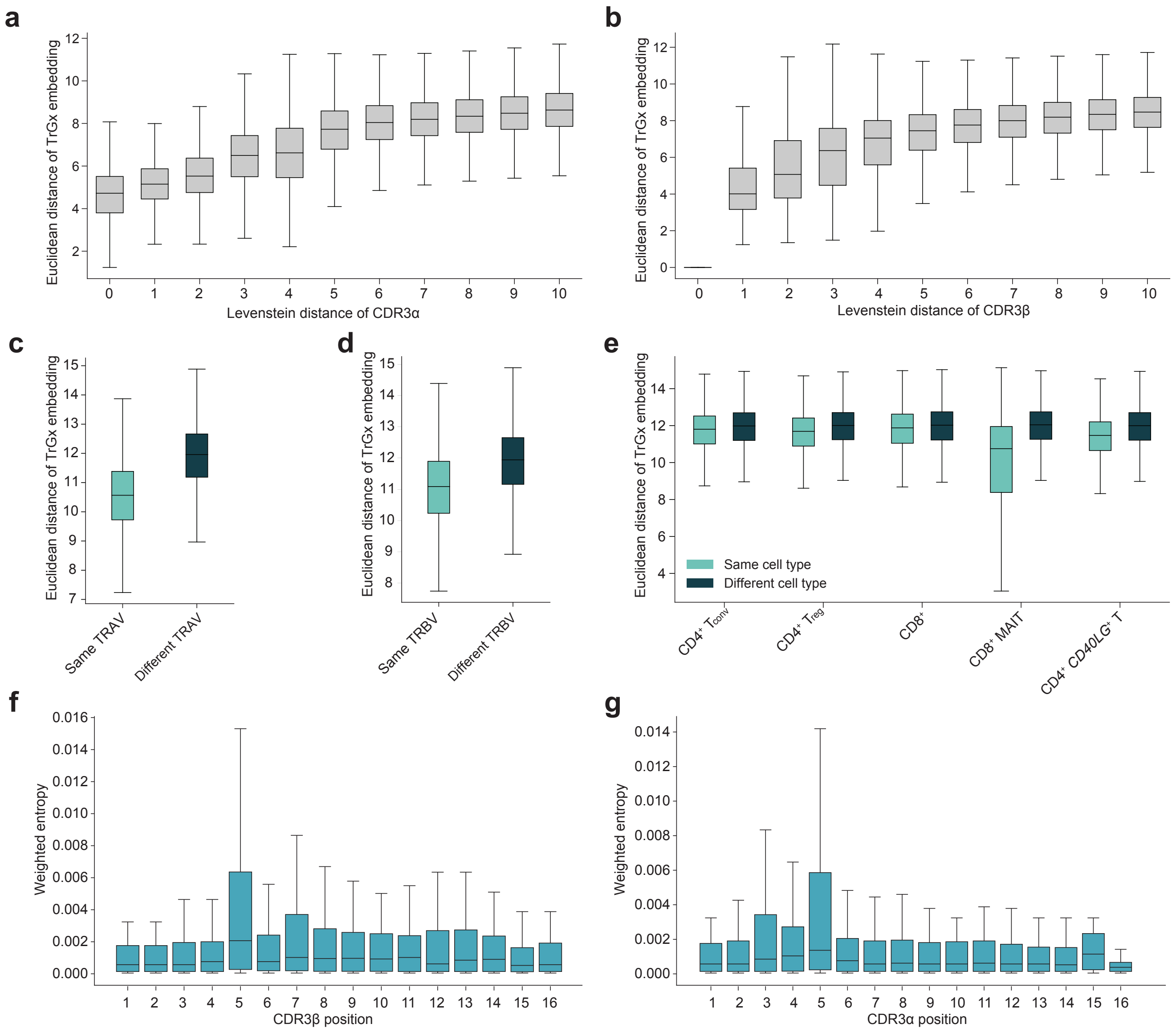

Supplementary Figure 6 | Analysis of Euclidean distances in TrGx embeddings and associated GEX and TCR features. **a**,**b**, Box plots displaying Euclidean distances within TrGx embeddings, stratified by Levenshtein distances between CDR3α and CDR3β sequences. **c**,**d**, Box plots showing Euclidean distances of TrGx embeddings based on whether two TCRs share the same TRAV (**c**) or TRBV (**d**) gene segments. **e**, Box plot illustrating Euclidean distances of TrGx embeddings categorized by whether TCRs are derived from cells belonging to the same or different major T cell subtypes. **f**,**g**, Weighted entropy of amino acid composition at each position of CDR3β (**f**) and CDR3α (**g**) for all cTrGx-TCRαβs using all public TCRαβ in Figure 2 as anchors. For all box plots, the middle lines represent the median; boxes represent the 25th (bottom) and 75th (top) percentiles; and whiskers represent the minimum and maximum points within 1.5× the interquartile range.

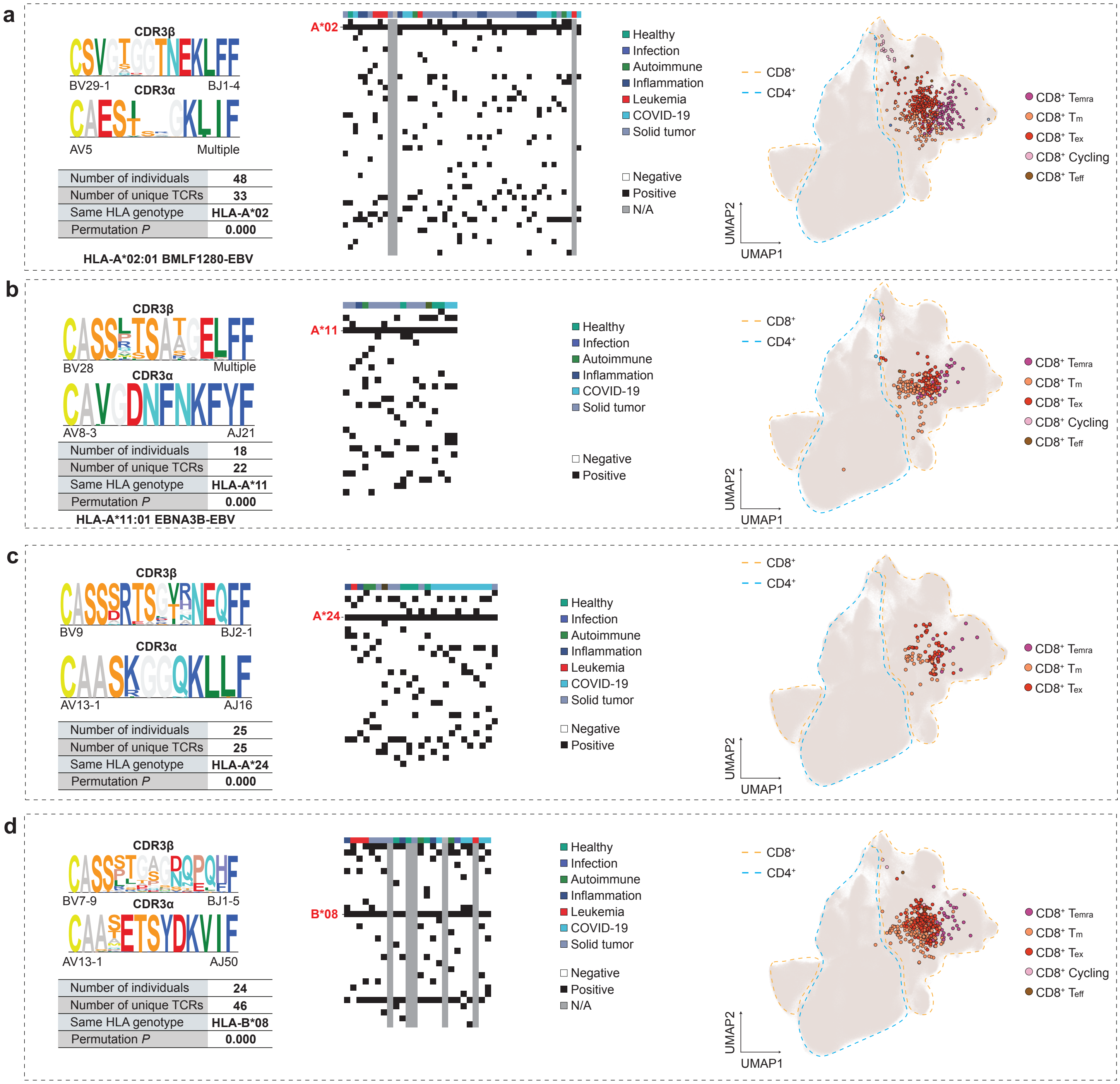

Supplementary Figure 7 | Searching for HLA-shared cGxTr-TCRαβ clonotypes. a–d, Additional representative cGxTr-TCRαβ clonotypes clustered by matching HLA-A*02 (**a**), HLA-A*11 (**b**), HLA-A*24 (**c**), and HLA-B*08 (**d**). For each cGxTr-TCRαβ cluster, the number of individuals, number of unique TCRαβs, matched HLA allele, and *p*-values from permutation tests are labeled. The binary heatmaps show the HLA genotypes from all individuals for each cluster. Motif plots of CDR3α and CDR3β from the cGxTr-TCRαβ clusters and the corresponding UMAP locations of these clusters are displayed.

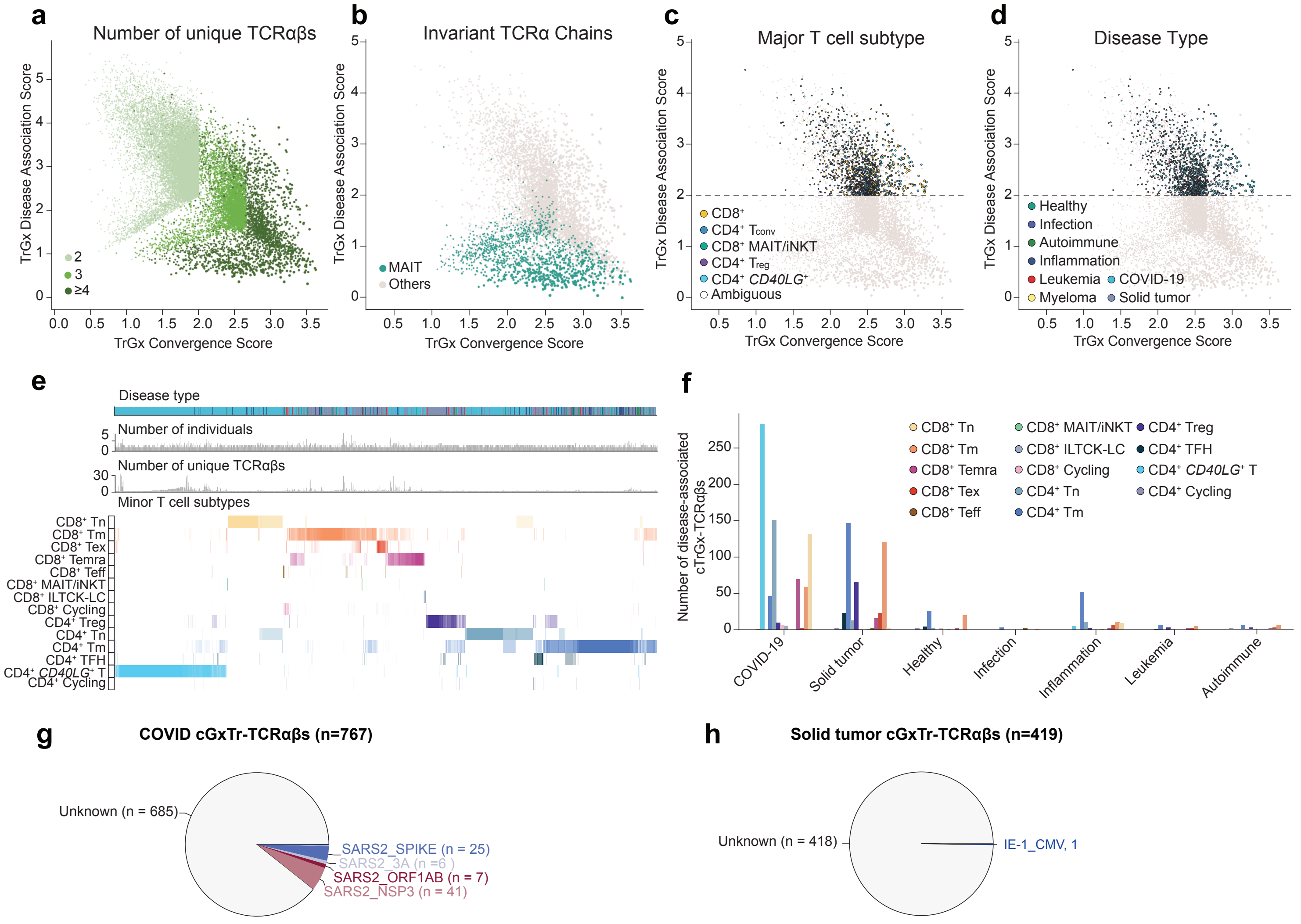

Supplementary Figure 8 | Additional information on the identification of disease-associated cGxTr-TCRαβs. **a**, Scatter plot of TrGx convergence score and disease association score of all cGxTr-TCRαβ clusters with unique disease type, colored by number of unique TCRαβs in each cluster. **b**, Scatter plot of TrGx convergence score and TrGx disease association score of cGxTr-TCRαβ clonotypes with more than 2 unique TCRαβs, colored by whether containing MAIT-associated invariant TCRα chains (**b**). **c**,**d**, Scatter plots of TrGx convergence scores and disease association scores for disease-associated cGxTr-TCRαβ clusters that have disease association scores greater than 2 and are derived from more than one individual, colored by major T cell subtype (**c**) and disease type (**d**). **e**, Summary plot of cGxTr-TCRαβ clonotypes with more than 2 unique TCRαβs, showing their disease type, number of individuals, number of unique TCRαβ clonotypes, and composition of minor T cell subtype. **f**, Bar plots of the number of cTrGx-TCRαβs grouped by disease types and colored by cell subtypes. **g**,**h**, Pie charts illustrating the composition of COVID-19- (**g**) and solid tumor-associated (**h**) cTrGx-TCRαβ clusters with at least one TCRαβ with known antigen specificity.

a

	incorporate GEX	CDR3β	TRBV	TRBJ	CDR3α	TRAV	TRAJ
TCR-DeepInsight	✔	✔	✔		✔	✔	
GLIPH2		✔	✔	✔	✔		
GIANA		✔	✔				
iSMART		✔	✔				
clusTCR		✔			✔		
TCRdist3		✔	✔	✔	✔	✔	✔
Tessa	✔	✔					
CoNGA	✔	✔	✔	✔	✔	✔	✔
scNAT	✔	✔	✔	✔	✔	✔	✔
mvTCR	✔	✔			✔		
MIST	✔	✔	✔	✔	✔	✔	✔

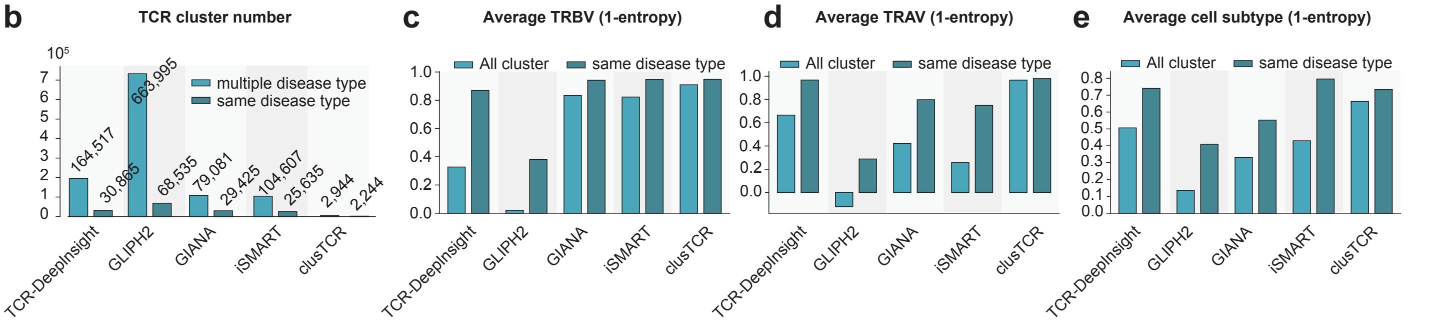

Supplementary Figure 9 | Comparison of TCR-DeepInsight with other methods. a, Summary table for methods in TCR repertoire analysis, where GLIPH2, GIANA, iSMART, and clusTCR are designed for data with TCR information only, and TCR-DeepInsight, Tessa, ConGA, scNAT, mvTCR, and MIST also consider GEX modality in single-cell immune profiling datasets. **b**, Bar plot of the number of clusters with multiple or same disease type by TCR-DeepInsight, GIANA, GLIPH2, and clusTCR on the pan-disease single-cell TCRαβ repertoire reference atlas. **c**–**e**, Bar plot of of averaged entropy of TRBV (**c**), TRAV (**d**), and cell subtype entropy (**e**) in clusters by TCR-DeepInsight, GIANA, GLIPH2, and clusTCR.

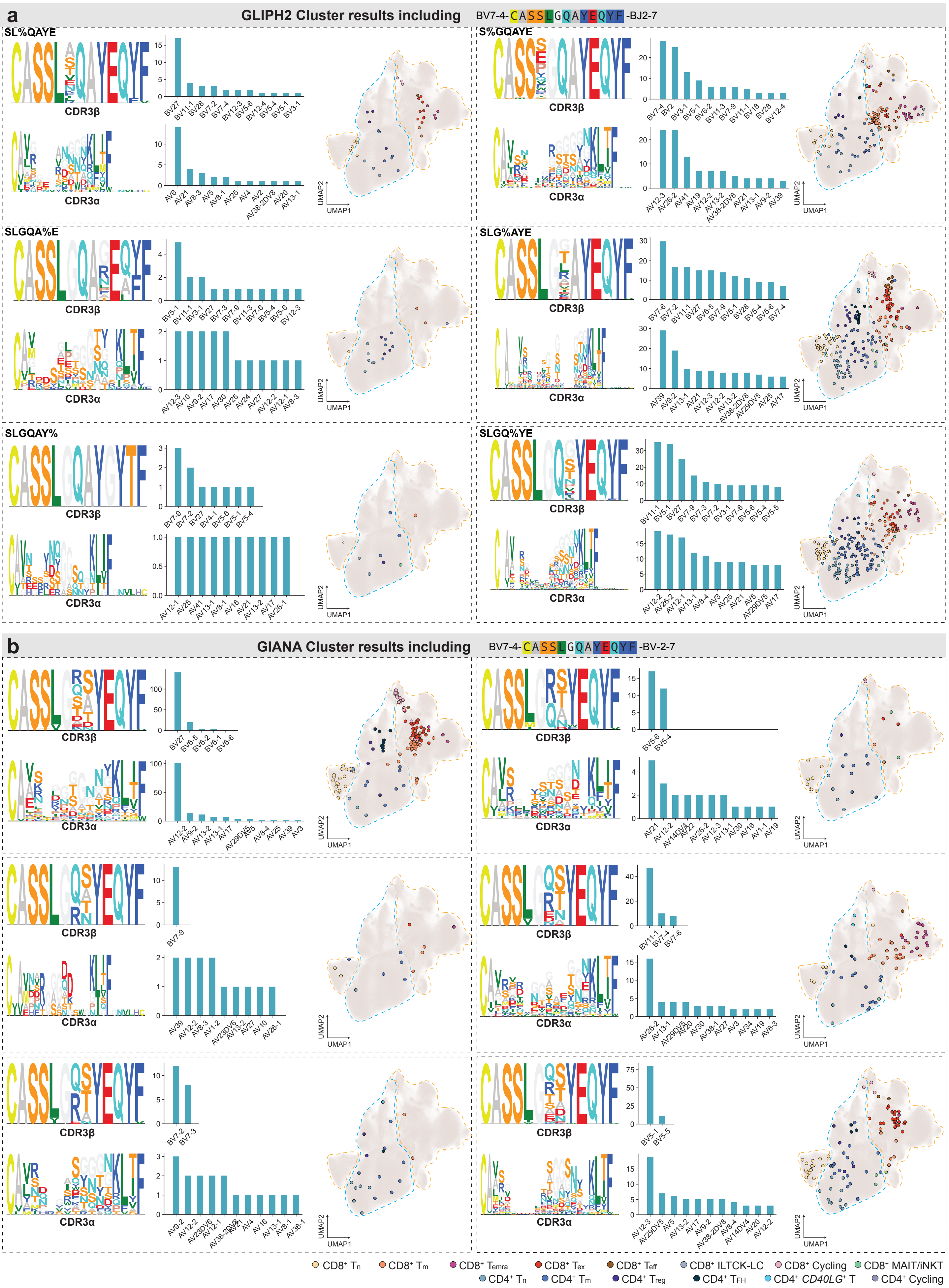

Supplementary Figure 10 | GLIPH2 and GIANA TCR clusters containing specific TCRβ chains.
a,**b**, Logo plot of amino acid sequences of CDR3β (upper left) and CDR3α (bottom left), bar plot of TRBV (upper middle) and TRAV usage (bottom middle), and UMAP plot highlighting cells within the TCR clusters (right) from GLIPH2 (**a**) and GIANA (**b**). The blue dashed lines outline the CD4+ T cells and the orange dashed lines outline the CD8+ T cells.

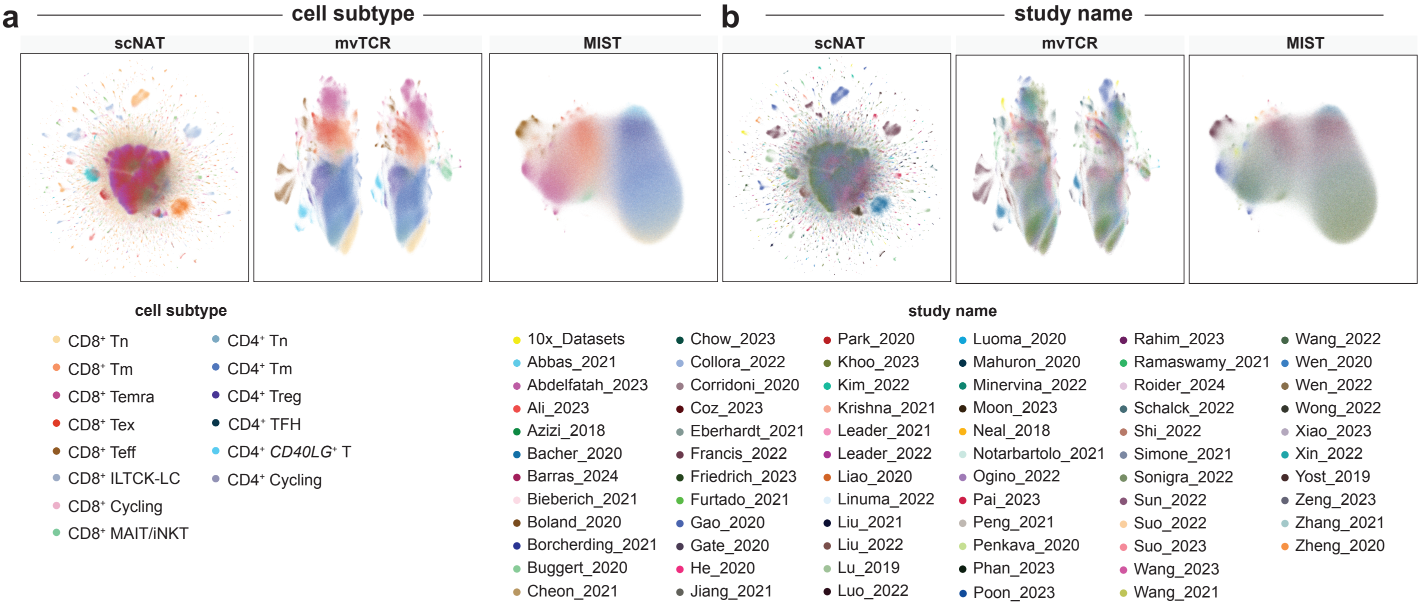

Supplementary Figure 11 | Joint GEX and TCR embeddings learned by scNAT, mvTCR, and MIST. a,**b**, UMAP of embedding learned by scNAT, mvTCR, and MIST, colored by cell subtypes (**a**) and study names (**b**).
